# Novel pyrazole carboxylate derivatives as lonazolac bioisosteres with selective COX-2 inhibition: design, synthesis and anti-inflammatory activity

**DOI:** 10.1007/s11030-025-11220-8

**Published:** 2025-05-22

**Authors:** Wael A. A. Fadaly, Ahmed Elshewy, Ahmed H. A. Abusabaa, Dina M. E. Amin, Hoda Khalifa Abdelhady, Haredy Hassan Haredy, Asmaa M. Mahmoud, Nashwa A. Ibrahim, Mohamed T. M. Nemr

**Affiliations:** 1 Pharmaceutical Organic Chemistry Department, Faculty of Pharmacy, Beni-Suef University, Beni-Suef, 62514 Egypt; 2 Pharmaceutical Organic Chemistry Department, Faculty of Pharmacy, Cairo University, Kasr El-Eini Street, Cairo, 11562 Egypt; 3 Department of Natural and Applied Sciences, College of Arts and Sciences, The American University of Iraq-Baghdad (AUIB), Baghdad, Iraq; 4 Department of Pharmaceutical Organic and Medicinal Chemistry, Faculty of Pharmacy, Fayoum University, Fayoum, 63514 Egypt; 5 Physiology Department, Faculty of Medicine, Al-Azhar University, Assuit, Egypt; 6 Pharmacology Department, Faculty of Medicine, Al-Azhar University, Assuit, Egypt; 7 Biochemistery Department, Faculty of Science, Beni-Suef University, Beni-Suef, 62511 Egypt; 8 Medicinal Chemistry Department, Faculty of Pharmacy, Beni-Suef University, Beni-Suef, 62514 Egypt

**Keywords:** Non-acidic Lonazolac, Celecoxib, COX-2, 5-LOX, 15-LOX, TNF-α, PGE2, iNOS

## Abstract

Two novel series of di-aryl/tri-aryl substituted pyrazole ester derivatives **15a-h** and** 19a-d** were designed, synthesized as novel non-acidic lonazolac analogs and tested for its COX-2, 5-LOX, 15-LOX, iNOS**,** pro-inflammatory cytokines TNF-α and PGE2 inhibitory activities. All the tested compounds showed excellent COX-2 inhibitory activity (IC_50_ = 0.059–3.89 μM), compared to that of celecoxib (IC_50_ = 0.22 μM), where derivatives **15c, 15d, 15 h** and** 19d** were found to be the most potent showing COX-2 selectivity index in range of (S.I. = 28.56–98.71) compared to celecoxib (S.I. = 13.65). Moreover, the most potent four derivatives **15c, 15d, 15 h** and** 19d** showed outstanding 5-LOX and 15-LOX inhibitory activities (IC_50_ = 0.24–0.81, 0.20–2.2 respectively, compared to zileuton IC_50_ = 1.52 and 0.54, respectively). Further investigation of the anti-inflammatory mechanistic study of derivatives **15c, 15d, 15 h** and** 19d** revealed that these four compounds exhibited comparable TNF-α and PGE2 (LPS-induced pro-inflammatory cytokines) inhibitory activities (IC_50_ = 0.77–1.20 μM and 0.28–0.52 μM respectively) when compared to celecoxib (IC_50_ = 0.87 μM and 0.38 μM respectively) as reference drug using lipopolysaccharide-activated RAW 264.7 macrophages. Based on the advanced inhibitory activity of compounds **15c, 15d, 15 h** and** 19d** against LPS-induced pro-inflammatory mediators (TNF-α and PGE2), inducible nitric oxide synthase (iNOS) inhibition assay was carried out. Remarkably, compounds **15c, 15d, 15 h** and** 19d** showed higher potency with lower IC_50_ (0.41–0.61 µM) when compared to the reference drug celecoxib (0.48 µM). Prior to in vivo anti-inflammatory activity screening, cytotoxicity testing was performed to ascertain safe and non-toxic concentrations of each compound. Safe doses of compounds were determined using lipopolysaccharide-activated RAW 264.7 macrophages, moreover results showed that compounds **15c, 15d, 15 h** and** 19d** were more safer (less cytotoxic) with higher IC_50_ (178.95–301.40 µM) when compared to the reference drug celecoxib (148.90 µM). In vivo anti-inflammatory activity of the target compounds **15c, 15d, 15 h** and** 19d** reinforced the results of in vitro screening as the derivatives **15c, 15d, 15 h** and** 19d** showed (ED_50_ = 8.22–31.22 mg/kg, respectively) and were more potent than celecoxib (ED_50_ = 40.39 mg/kg). All screened derivatives **15c, 15d, 15 h** and** 19d** were less ulcerogenic (ulcer indexes = 1.22–3.93) than lonazolac (ulcer index = 20.30) and comparable to celecoxib (ulcer index = 3.02). In silico docking and ADME studies were carried out in order to clarify the interactions of the most active derivatives **15c, 15d, 15 h** and** 19d** with the target enzymes and their pharmacokinetic parameters.

## Introduction

Inflammation is the body tangled protective mechanism against any pathogens, injuries, or bacterial invasion [1]. This mechanism is characterized by swelling, redness and pain associated with vascular and cellular alterations [2]. It is a complex process in which many enzymes and mediators are involved such as inducible NO synthetase (iNOS), 5-LOX, 15-LOX and COX-2 that leads to development of inflammation-related diseases, such as cardiovascular disorders and cancer [3, 4].

The first stage in the inflammatory response is the release of pro-inflammatory mediators (e.g., prostaglandins, histamine, leukotrienes and inducible nitric oxide NO), this leads to vasodilation, as a consequence, a series of biochemical proceedings and passage of leukocytes occurs from blood to the affected tissue [5].

Non-steroidal anti-inflammatory drugs (NSAIDs) are among the most commonly used drugs due to their anti-inflammatory, antipyretic and analgesic actions [6]. The anti-inflammatory activity of NSAIDs is due to their ability to inhibit cyclooxygenase (COX)-mediated production of pro-inflammatory mediators like; prostaglandins (PGs), cytokines tumor necrosis factor (TNF-α), interleukin-1 (IL-1), interleukin-2 (IL-2), interleukin-6 (IL-6) and thromboxanes (TXs) [7]. Additionally, lipopolysaccharide (LPS) is one of the essential elements promote macrophage activation, causing the release of transcription factors and pro-inflammatory mediators [8]. Controlling the NO and PGE2 generation in LPS-stimulated macrophages is therefore thought to be a great model for investigating of the effectiveness of the anti-inflammatory drugs [9, 10].

On the other hand, lipoxygenases (LOXs) are a non-heme iron-containing dioxygenases that catalyze the synthesis of leukotrienes from arachidonic acid and other unsaturated fatty acids by adding molecular oxygen (O_2_) in the form of a hydro-peroxyl (HO_2_) residue [11, 12]. From literature survey, 5-lipoxygenase (5-LOX) has been escorting to disorders like asthma, chronic bronchitis, rheumatoid arthritis and cancer through controlling LOX pathway which is mediated by inflammation and hyper-proliferation [13–15]. Furthermore, inducible-NOS (iNOS) are one of the nitric oxide synthase isoforms that can be stimulated by bacterial lipopolysaccharide (LPS) or cytokines such as TNF-α. Inducible-NOS produce large, cytotoxic amounts of NO that can mediate inflammation and innate immune response [16–18].

Many manuscripts revealed that, Coxibs containing the bioactive pharmacophores, either (SO_2_NH_2_) as in **celecoxib (1)** with pyrazole scaffold or (SO_2_CH_3_) as in **rofecoxib (2)** with furanone as central ring are characterized by the Y-shaped structural design and considered as selective COX-2 inhibitors with the most brilliant scaffolds displaying advanced anti-inflammatory activity [19–22]. **Lonazolac (3)** is 1,3-disubstituted-pyrazole acetic acid drug, with potent analgesic and anti-inflammatory activities, but is non-selective COX-2 inhibitor so it associated with gastrointestinal side effects like other traditional NSAIDs.

In 2018, it was reported by our team through some structural modifications of **lonazolac (3),** the synthesis of non-acidic 1,3,4-trisubstituted-pyrazole derivatives as lonazolac analogs **(4a-l)** [19]. Additionally, the pyrazolyl oxime derivative **(5)** was designed, synthesized and tested for anti-inflammatory activities; it was found to be more selective for COX-2 isozyme in comparison to **celecoxib (1)** [23]. Previously, synthesis of 1,5-diarylpyrazole carboxylic acid **(6)** was reported, which had higher COX-2 selectivity index (S.I. = 2.94) compared to that of celecoxib (S.I. = 7.70) [24].

Furthermore, a novel series of fluorinated triaryl-pyrazoles **(7a-c)** was reported to be with advanced anti-inflammatory activity at all-time intervals (% edema inhibition = 42.1–87.9) with better gastric profile (ulcer index U.I. = 1.25–2.5) than the traditional NSAID; indomethacin (ulcer index U.I. = 14) and were close to the selective COX-2 inhibitor; celecoxib (ulcer index U.I. = 1.75) [25]. Moreover, compounds **(8a**) (IC_50_ = 0.67 μM) and **(8b)** (IC_50_ = 0.58 μM) showed better COX-2 inhibitory activity than celecoxib (IC_50_ = 0.87 μM) with selectivity index (S.I. = 8.41 and 10.55, respectively) relative to celecoxib (S.I. = 8.85) [26]. As well as, compounds **(8a** and** 8b)** exhibited advanced inhibitory activity against 5-LOX (IC_50_ = 1.92, 2.31 μM) higher than zileuton as reference drug (IC_50_ = 2.43 μM) [26].

**Fig. 1:**
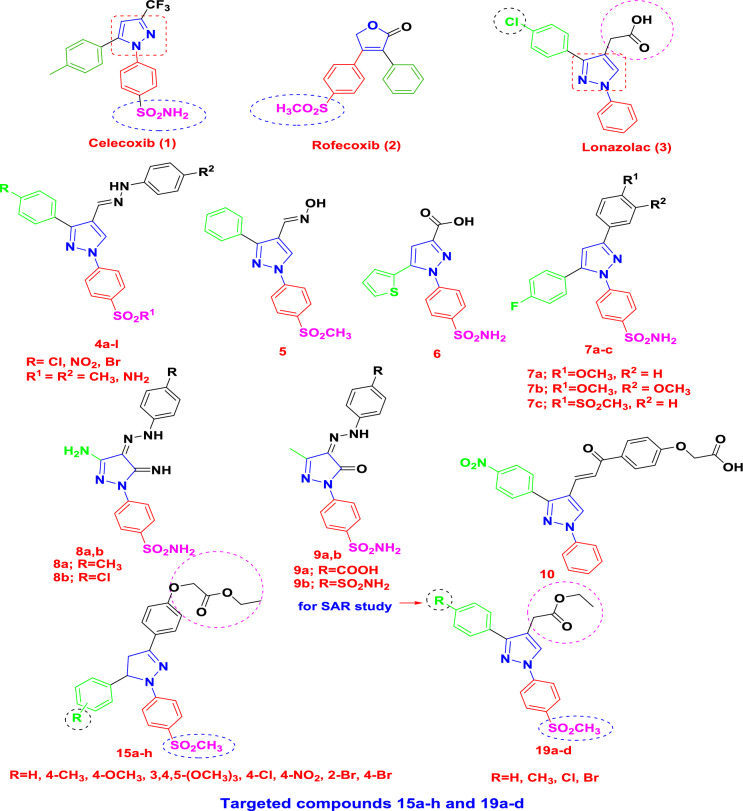
Selective COX-2 inhibitors **(rofecoxib (1), celecoxib (2), lonazolac (3),** reported derivatives** 4–10** and rationale design of the target compounds **15a-h** and** 19a-d**

In addition, compounds **(9a** and **9b)** were found to be the most potent COX-2/5-LOX inhibitors compared to standard celecoxib (S.I. = 3.52) [27]. Finally, pyrazole containing acid derivative **(10)** exhibited promising inhibitory activity towards 5-LOX (IC_50_ = 5.88 µM), the anti-inflammatory activity of this compound was confirmed by high iNOS and PGE2 inhibitory activities in LPS-stimulated RAW cells with IC_50_ values of 4.93 µM and 10.98 µM, respectively [28] (Fig. 1).

Synthesis of highly effective and enhanced safety-profiled anti-inflammatory drugs has turned out to be a stimulating chore in the drug innovation process. Though several improvements have been made, there is still essential need to invent more effective anti-inflammatory drugs with minimal side effects [29]. Based on the aforementioned data and in continuation of our research specialized with the design, synthesis and anti-inflammatory assessment of **celecoxib** and **lonazolac** analogs [30–43], we have proposed the synthesis of new hybrid mimic structures of triaryl and diaryl-pyrazole derivatives **(15a-h)** and **(19a-d)** with an ester substituent instead of carboxylic acid moiety of lonazolac to decrease the local ulceration side effect and investigated their anti-inflammatory properties and COX-2 selectivity inhibition through incorporation of bioactive pharmacophore, Y-shaped design with central pyrazole containing (SO_2_CH_3_) moiety.

## Rationale and structure-based design

Our idea in the rationale of the target derivatives **15a-h** and** 19a-d** comes from that, these derivatives exposed the essential pharmacophoric features of COX-2 inhibitors, through bioisosteric modification strategies of selective COX-2 inhibitors **(celecoxib (1)**, **rofecoxib (2)), lonazolac (3),** non-acidic 1,3,4-trisubstituted-pyrazole derivatives **4a-l** and Y-shaped pyrazole containing derivatives **5–10**. As COX-2 inhibitors, **i)** pyrazole central ring in the target compounds was maintained, resembles that in **celecoxib (1)** and** lonazolac (3)**. **ii)** The essential pharmacophore SO_2_NH_2_ in **celecoxib (1)** was replaced with the other active one, SO_2_CH_3_ as in **rofecoxib (2)** which is significant for enhancing COX-2 selectivity of our designed lonazolac analogues. **iii)** Moreover, the 4-chlorophenyl moiety at pyrazole C-3 of **lonazolac (3)** was maintained or replaced with another electronegative moiety as 4-nitrophenyl, 2-bromophenyl and 4-bromophenyl (as in our previously reported derivatives **4a-l**) or electron-donating groups like 4-toloyl, 4-methoxyphenyl and 3,4,5-trimethoxyphenyl in the target compounds **15a-h** and **19a-d** to study the effect of these different groups on COX-2 selectivity. **iv)** Acetic acid moiety in position-4 of the pyrazole nucleus of **lonazolac (3)** or the more bulky moiety (methane/aminosulfonylphenylhydrazinomethene) in position-4 of its previously reported analogue, pyrazole derivatives **(4a-l)** was replaced with either steric arm (phenoxyacetic acid ester) as in target compounds **15a-h** or (ethyl carboxylate moiety) as in target derivatives **19a-d**, these replacements are supposed to reduce acidity of target compounds in order to have safer gastric profile. **v)** In addition, triaryl-substitution of pyrazoline nucleus was carried out as in target compounds **15a-h** in order to resemble that of previously reported compounds **(7a-c)** (excellent anti-inflammatory potency). **vi)** Furthermore, the phenylhydrazone group in position-4 of the pyrazole nucleus of compounds** 8a, 8b, 9a** and** 9b** was replaced with the steric arm (phenoxyacetic acid ethyl ester) as in target compounds **15a-h**. **vii)** Finally, the presence of the carboxylic group or its pro-ester function as in derivative **(10),** which is essential for 5-LOX and iNOs activities; was maintained in the target derivatives **19a-d** in form of ester moiety. All these modifications prompted us to study the structure activity relationship of the target compounds **15a-h** and** 19a-d** as anti-inflammatory compounds.

## Results and discussion

### Chemistry

#### Schemes for synthesis of the final target compounds 15a-h and 19a-d

**Scheme 1:**
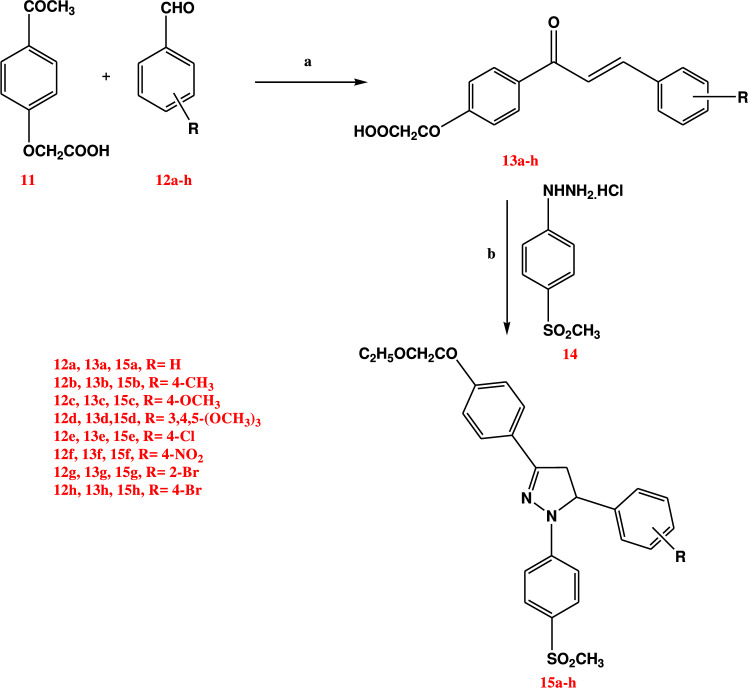
Reagents and conditions: **a** NaOH, EtOH (95%), stirring at room temp., 24 h; **b** EtOH (95%), reflux, 36 h

In scheme 1, the general reactions used for the preparation of the final target ethyl pyrazolyl-ester derivatives **15a-h** were outlined. The chalcone acetic acid derivatives **13a-h** were obtained using 4-acetylphenoxyacetic acid **11** that prepared according to reported procedure [44] and various aromatic aldehydes **12a-h** as starting materials, according to the literature through a base-catalyzed Claisen–Schmidt condensation at room temperature [44–46]. Furthermore, cyclo-condensation of the appropriate chalcone acetic acid derivatives **13a-h** with 4-methanesulfonylphenylhydrazine hydrochloride **14** [47, 48] in aqueous ethanol afforded the respective 1,3,5-triaryl-4,5-dihydro-1*H*-pyrazole **15a-h** in good yields (61–88%). The prepared compounds have been characterized by IR, ^1^H NMR, DEPTQ-^13^C NMR spectra and elemental analyses, the IR spectra of compounds **15a-h** showed a sharp peak at 1755–1732 cm^−1^ corresponding to the ester C = O group, two sharp peaks at 1300–1400 cm^−1^ and 1130–1141 cm^−1^ corresponding to SO_2_CH_3_ group. ^1^H NMR spectra of **15a-h** revealed the presence of three signals as a doublet of doublet (dd) each of one proton intensity, one at δ 3.11–3.22 ppm, the second at δ 3.89–4.03 ppm and the third at δ 5.54–5.89 ppm with three different *J* values (17.6 Hz, 12.0 Hz, 4.8–5.2 Hz) corresponding to three protons of the 4,5-di-hydropyrazole ring. The highest *J* value was due to the geminal coupling of two protons at position 4, while the other two *J* values were due to the coupling of two geminal protons with the vicinal proton at position-5. Likewise, DEPTQ-^13^C NMR of the pyrazoline derivatives **15a-h** revealed signals at δ 61.20–62.66 ppm and at δ 42.53–44.63 ppm indicating the CH, CH_2_ of the pyrazoline ring respectively (Scheme 1).

An interesting formation of the ethyl ester derivatives **15a-h** on the expense of acid derivatives was proved by the careful investigation of ^1^H NMR and IR spectra for these compounds **15a-h** as in IR spectra, the dragged carboxylic peak was unobserved in pyrazolyl ester derivatives **15a-h** but only the appearance of sharp carbonyl group peak at 1755–1732 cm^−1^. In addition, ^1^H NMR spectra showed the characteristic signals of ethyl ester group in all derivatives **15a-h** as triplet and quartet peaks at δ 1.20–1.23 ppm and at 4.15 ppm respectively. Moreover, DEPTQ-^13^C NMR of the pyrazoline derivatives **15a-h** revealed signals at δ 14.49–14.52 ppm and at δ 65.15–65.19 ppm indicating the CH_3_, CH_2_ of the ester group respectively. Formation of ethyl ester derivatives on expense of acid derivatives was contributed to the Fisher esterification of acidic group of derivatives **13a-h** by ethanol that used as solvent and with the aid of the hydrochloride of 4-methanesulfonylphenylhydrazine hydrochloride **14** during cyclization of these chalcone derivatives **13a-h** into the pyrazole derivatives **15a-h.**

**Scheme 2:**
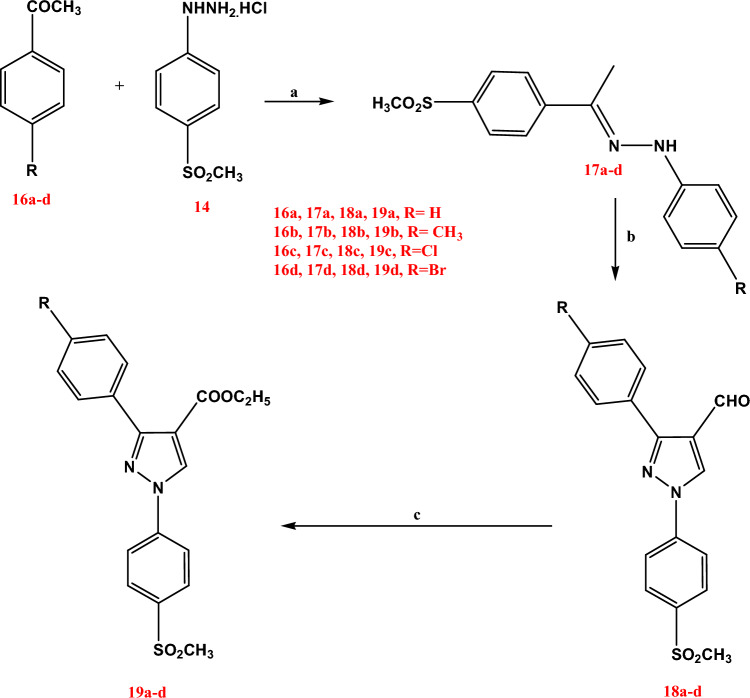
Reagents and conditions: **a** ethanol (95%), reflux, 24 h; **b** DMF, POCl_3_, reflux, 24 h; **c** Oxone, ethanol (95%), stirring at room temp., 24 h

In scheme 2, the reaction pathways for the synthesis of the diaryl-pyrazolyl ester derivatives **19a-d** were outlined. A one-pot reaction using an ethanolic solution of oxone is used to synthesize the ester derivatives **19a-d** from the corresponding aldehydes **18a-d** that were previously prepared through Vilsiemier Haack reaction conditions of their preliminary hydrazone derivatives **17a-d** [19]. The ester formation resulted from the oxidation of aldehyde to carboxylic acid followed by Fischer-type esterification of the acids in alcoholic solvent was illustrated by IR, ^1^H NMR, DEPTQ-^13^C NMR spectra and elemental analyses, IR spectra showed a characteristic absorption band at 1701–1693 cm^−1^ attributed to the carbonyl group of ethyl ester moiety. As well, ^1^H NMR spectra demonstrated a singlet signal at δ 1.26 ppm and at δ 4.22–4.23 ppm corresponding to CH_3_, CH_2_ of the ester group respectively. Also, DEPTQ-^13^C NMR of the pyrazoline derivatives **19a-d** revealed signals at δ 14.56–14.60 ppm and at δ 60.79–60.62 ppm indicating the CH_3_, CH_2_ of the ethyl ester functionality (Scheme 2).

## Biological evaluation

### In vitro anti-inflammatory activity against COX-1 and COX-2 enzymes

The in vitro COX-1/COX-2 inhibition was assessed using the COX-1 Inhibitor Screening Kit-K548 and the COX-2 Inhibitor Screening Kit-K547 from Biovision, S. Milpitas Blvd., Milpitas, CA 95035 USA [29]. The results showed that compounds **15c, 15d, 15 h** and** 19d** have strong inhibitory activity against COX-2 isozyme (IC_50_ = 0.059–3.89 μM), compared to that of celecoxib (IC_50_ = 0.22 μM), where derivatives **15c, 15d, 15 h** and** 19d** were found to be the most potent showing COX-2 selectivity index in range of (28.56–98.71) compared to celecoxib (S.I. = 13.65) (Table 1).

**Table 1:** In vitro COX-1 and COX-2 inhibitory activities of derivatives **15a-h, 19a-d** and the reference celecoxib

Compound	COX-1 IC_50_ μM^a^	COX-2 IC_50_ μM^a^	COX-2 S.I.^b^
15a	7.422 ± 0.45	3.897 ± 0.129	1.90
15b	11.42 ± 0.42	1.94 ± 0.064	5.89
15c	11.341 ± 0.45	0.397 ± 0.013	28.56
15d	15.519 ± 0.61	0.221 ± 0.007	70.22
15e	3.006 ± 0.12	0.742 ± 0.025	4.05
15f	6.826 ± 0.25	1.586 ± 0.052	4.30
15 g	8.589 ± 0.33	0.575 ± 0.019	15.07
15 h	6.884 ± 0.25	0.103 ± 0.003	66.83
19a	14.561 ± 0.53	2.33 ± 0.077	6.24
19b	9.417 ± 0.4	2.473 ± 0.100	3.80
19c	18.070 ± 0.66	2.005 ± 0.080	9.01
19d	5.824 ± 0.23	0.059 ± 0.002	98.71
Celecoxib	3.005 ± 0.12	0.22 ± 0.007	13.65

^a^The concentration of test compound produce 50% inhibition of COX-1, COX-2 enzyme, the result is the mean of six values ± standard deviation. ^b^The in vitro COX-2 selectivity index (COX-1 IC_50_/COX-2 IC_50_)

### In vitro anti-inflammatory activity against 5-LOX/15-LOX enzymes

The in vitro 5-LOX/15-LOX inhibition was assessed using the 5-Lipoxygenase Inhibitor Screening Kit (Catalog # K980-100) from Biovision, S. Milpitas Blvd., Milpitas, CA 95035 USA and the Lipoxygenase Inhibitor Screening assay Kit (Item # 760,700) from Cayman Chemical, 1180, East Ellsworth Rd., Ann Arbor, MI 48108, USA [49, 50]. The test compounds potency was revealed by calculating IC_50_. The results exhibited that compounds **15c, 15d, 15 h** and** 19d** had excellent inhibitory action against 5-LOX isozyme (IC_50_ = 0.24–0.81 μM range) and enhanced inhibitory activity against 15-LOX isozyme (IC_50_ = 0.20–2.2 μM range) compared to zileuton IC_50_ = 1.52 and 0.54, respectively) (Table 2).

**Table 2:** In vitro 5-LOX/15-LOX, TNF-α, PGE2 and iNOS production and cytotoxicity in LPS-activated RAW 264.7 macrophages inhibitory activities of derivatives **15c, 15d, 15 h, 19d** and reference compounds (Zileuton and celecoxib)

Compound	5-LOX IC_50_ μM^a^	15-LOX IC_50_ μM^a^	TNF-α IC_50_ μM^a^	PGE2 IC_50_ μM^a^	iNOS IC_50_ μM^a^	Cytotoxicity IC_50_ μM (LPS-induced RAW 264.7 cells)
15c	0.453 ± 0.018	0.412 ± 0.016	0.77 ± 0.025	0.52 ± 0.029	0.410 ± 0.06	301.40 ± 10.7
15d	0.241 ± 0.009	2.232 ± 0.087	1.20 ± 0.060	0.33 ± 0.021	0.531 ± 0.05	182.99 ± 6.52
15 h	0.813 ± 0.032	0.488 ± 0.019	1.04 ± 0.042	0.28 ± 0.032	0.610 ± 0.13	178.95 ± 6.37
19d	0.337 ± 0.013	0.208 ± 0.008	1.01 ± 0.041	0.45 ± 0.026	0.481 ± 0.12	209.45 ± 7.46
Celecoxib	––-	––-	0.87 ± 0.030	0.38 ± 0.027	0.480 ± 0.04	148.90 ± 5.30
Zileuton	1.524 ± 0.059	0.542 ± 0.021	––-	––-	––-	–––

^a^The concentration of test compound produce 50% inhibition, the result is the mean of six values ± standard deviation

#### In vitro anti-inflammatory mechanistic study of compounds **15c, 15d, 15 h** and **19d** against pro-inflammatory mediators TNF-α and PGE2 cytokines production in LPS-activated RAW 264.7 macrophages

Further investigation of the anti-inflammatory mechanistic study of compounds **15c, 15d, 15 h** and** 19d** via assessing their inhibitory action against LPS-induced pro-inflammatory cytokines (TNF-α and PGE2) production. The results were summarized in Table 2. The results revealed that compounds **15c, 15d, 15 h** and** 19d** exhibited excellent TNF-α and PGE2 inhibitory activities (IC_50_ = 0.77–1.20 μM and 0.28–0.52 μM respectively) when compared to celecoxib (IC_50_ = 0.87 μM and 0.38 μM respectively) as a reference drug (Table 2).

### Inducible nitric oxide synthase (iNOS) inhibition

In LPS-induced RAW 264.7 cells, the inhibitory effects of compounds **15c, 15d, 15 h** and** 19d** on iNOS activity production were investigated (Table 2). When tested on LPS-induced RAW 264.7 cells, the active four compounds showed a significant inhibitory effect on iNOS (0.41–0.61 µM) when compared to the reference drug celecoxib (0.48 µM).

### Cytotoxicity determination of the target compounds

Prior to assessing the in vivo anti-inflammatory activity, the MTS assay was used to evaluate the cytotoxicity of the most potent synthesized compounds **15c, 15d, 15 h** and** 19d** against RAW 264.7 macrophages [36–42]. None of the tested compounds demonstrated any noticeable cytotoxicity and showed high IC_50_ concentrations (Table 2). Therefore, the in vivo anti-inflammatory activity of these derivatives was further assessed.

### In vivo anti-inflammatory activity

Using a dose of 50 mg/kg body weight, the most selective COX-2 inhibitors, **15c, 15d, 15 h** and** 19d** as well as celecoxib as a reference drug, were evaluated for their in vivo anti-inflammatory efficacy using the carrageenan-induced rat paw edema assay, in accordance with the reported procedure [35]. The anti-inflammatory activity was determined using paw-thickness changes at 1 h, 3 h, and 5 h following carrageenan injection, as shown in (Table 3). When compared to carrageenan, the most potent compounds **15c, 15d, 15 h** and** 19d** were found to significantly reduce inflammation at all times. Comparable studies comparing the tested compounds’ anti-inflammatory activity to celecoxib's at different time intervals found that, after 1 h, the tested compounds had good anti-inflammatory activity (A.I. = 38.3–61.1%) compared to celecoxib's (A.I. = 30.1%). All compounds showed increased anti-inflammatory activity after 3 h (A.I. = 41.9–82.0%). In a similar way, all compounds showed increased anti-inflammatory effect after 5 h (A.I. = 40.0–92.2%). Furthermore, in comparison to celecoxib, the most effective anti-inflammatory derivatives **15c, 15d, 15 h** and** 19d** had their dose inducing 50% edema inhibition (ED_50_) evaluated. Derivatives **15c, 15d, 15 h** and** 19d** (ED_50_ values of 8.22–31.22 mg/kg) were more potent than celecoxib (ED_50_ values of 40.39 mg/kg). (Table 4).

**Table 3:** In vivo anti-inflammatory activity of derivatives **15c, 15d, 15 h, 19d** and celecoxib

Compound No	Paw edema thickness (mm) ± SEM (% inhibition)		
	1 h	3 h	5 h
15c	3.1 ± 0.002 (38.3%)	2.56 ± 0.0034 (55%)	2.1 ± 0.0084 (66.1%)
15d	2.23 ± 0.0045 (61.1%)	2.79 ± 0.011 (41.9%)	2.90 ± 0.002 (40%)
15 h	2.57 ± 0.0071 (54.85%)	2.61 ± 0.018 (55.9%)	2.44 ± 0.0099 (59.3%)
19d	2.85 ± 0.025 (40.7%)	1.3 ± 0.006 (82%)	0.21 ± 0.0094 (92.2%)
Celecoxib	3.43 ± 0.004 (30.1%)	3.1 ± 0.011 (38.3%)	2.52 ± 0.0095 (56.3%)

Values represent means ± SEM of four animals for each group

**Means significant difference with celecoxib at p < 0.05

***Means highly significant difference with celecoxib at p < 0.005

**Table 4:** **ED**_**50**_ for the most active derivatives **15c, 15d, 15 h, 19d** and reference drug celecoxib

Compound No	% inhibition			
	10 mg/kg	25 mg/kg	50 mg/kg	ED_50_ (mg/kg)^a^
15c	22.32%	31.51%	70.02%	24.1
15d	29.52%	36.61%	80.29%	31.22
15 h	19.49%	37.98%	82.28%	29.9
19d	35.11%	52.23%	74.99%	8.22
Celecoxib	55.48%	34.97%	97.48%	40.39

^a^The concentration of test compound produce 50% edema inhibition

### Ulcerogenic liability

The most potent compounds **(15c**,** 15d**,** 15 h** and** 19d)** were subjected to further evaluation to determine their ulcerogenic effect (ulcer index) [51] in comparison with celecoxib and lonazolc using 50 mg/kg dose. The results revealed that all compounds were less ulcerogenic (ulcer indexes = 1.22–2.93) than lonazolac (ulcer index = 20.30) and comparable to celecoxib (ulcer index = 3.02) which greatly supported our main objective to avoid gastric ulceration caused by COX-1 inhibition (Table 8). The diaryl pyrazole ester derivative (**19d**) was the most safe derivative (ulcer index = 1.22) which is about 20-fold less ulcerogenic than lonazolac and showed in our assay an ulcerogenic potential less than that of celecoxib (Table 5).

**Table 5:** Ulcer index for the most potent derivatives **15c, 15d, 15 h, 19d**, reference drugs celecoxib and lonazolac

Compound	Average severity	Average no of ulcer^a^	% incidence /10	Ulcer index
15c	0.17 ± 0.004***^b^	0.55 ± 0.012***^b^	2	2.61
15d	0.51 ± 0.013^b^	0.47 ± 0.006^b^	2	2.93
15 h	0.29 ± 0.009***^b^	0.40 ± 0.006^b^	2	2.60
19d	0.15 ± 0.002***^b^	0.12 ± 0.003***^b^	1	1.22
Celecoxib	0.66 ± 0.026***^b^	0.48 ± 0.015***^b^	2	3.02
Lonazolac	2.29 ± 0.13	8.1	10	20.30

^a^Values represent means ± SEM of ten animals for each group

***Means significant difference with celecoxib at p < 0.001

^b^Means significant difference with lonazolac at p < 0.001

## Molecular modeling studies

In order to investigate the possible binding mode of the synthesized compounds with either COX-2 or 5-LOX receptors, molecular docking study was performed using Molecular Operating Environment (MOE) version 2015.10 modeling software. The X-ray, crystal structure data for the enzymes were obtained from the protein data bank with code (PDB ID: 3LN1) [52] and (PDB ID: 3V99) [53] respectively. Docking scores, amino acid residues forming hydrogen bonding interactions and their lengths were summarized in Tables 6 and 7.

**Table 6:** Molecular data for compounds **15a-h**, **19a-d** and celecoxib during docking inside COX-2 receptor

Compound No	E-score Kcal/mol	No of hydrogen bonds	Hydrogen bonding residues	Hydrogen bonding type	Distance (A^o^)
15a	−12.90	1	Ser339	Arene-H	4.00
15b	−16.43	2	Ser339 Ala513	H-donor Arene-H	3.25 3.67
15c	−17.64	2	Arg449 Ala513	H-accept Arene-H	2.74 3.86
15d	−17.64	2	Ser339 Ser339	H-donor Arene-H	3.18 3.93
15e	−16.74	2	Arg449 Glu510	H-accept H-donor	2.73 3.13
15f	−16.44	2	Ser339 Ser516	Arene-H H-accept	3.88 3.10
15 g	−17.30	2	Arg449 Ala513	H-accept Arene-H	2.92 4.33
15 h	−17.67	2	Ser339 Arg449	Arene-H H-accept	3.66 2.73
19a	−13.57	1	Ser339	Arene-H	3.82
19b	−13.53	2	Ser339	Arene-H	3.78
19c	−15.67	1	Arg449	H-accept	2.89
19d	−18.17	5	Leu338 Arg449 Ser339 Ser516 Gln178	Arene-H H-accept Arene-H H-accept H-donor	4.21 2.71 3.99 3.00 3.46
Celecoxib	−13.57	4	Arg449 Leu338 Ser339 Gln178	H-accept H-donor H-donor H-donor	3.53 3.04 2.93 3.06

**Table 7:** Molecular data for compounds **15c**, **15d, 15 h, 19d,** zileuton and celecoxib during docking inside **5-LOX** receptor

Compound No	E-score Kcal/mol	No of hydrogen bonds	Hydrogen bonding residues	Hydrogen bonding type	Distance (A^o^)
15c	−16.25	1 + 7 with HOH	Gly50 HOH 393 HOH 356 HOH 338 HOH 459 HOH 343 HOH 459 HOH 595	H-accept H-accept Arene-H H-accept H-accept H-accept H-accept H-accept	3.42 3.26 3.52 3.23 3.10 2.91 3.13 3.00
15d	−11.78	3 + 7 with HOH	Gly50 Lys67 Glu121 HOH 393 HOH 357 HOH 475 HOH 352 HOH 577 HOH 338 HOH 459	H-accept H-accept H-donor H-accept Arene-H H-donor H-accept H-donor H-accept H-accept	3.54 2.99 2.74 3.50 3.74 3.64 2.81 3.68 3.32 3.26
15 h	−14.17	1 + 5 with HOH	Lys67 HOH 499 HOH 352 HOH 352 HOH 475 HOH 596	H-accept H-accept H-accept H-accept H-accept H-accept	2.83 3.47 2.83 3.52 3.57 3.16
19d	−18.17	2 + 10 with HOH	Gly50 Lys67 HOH 543 HOH 596 HOH 336 HOH 338 HOH 459 HOH 462 HOH 352 HOH 412 HOH 462 HOH 543	H-accept H-accept H-donor H-donor H-accept H-accept H-accept H-accept H-accept H-accept H-accept H-accept	3.05 3.09 3.23 3.13 3.57 2.83 2.81 3.49 2.58 2.83 2.72 3.33
Zileuton	−12.00	1 + 8 with HOH	Gly50 HOH 393 HOH 356 HOH 475 HOH 577 HOH 343 HOH 393 HOH 459 HOH 356	H-accept H-donor H-donor H-donor H-donor H-accept H-accept H-accept Arene-H	3.60 3.30 2.83 3.36 3.42 3.22 3.04 3.30 3.77
Celecoxib	−13.41	2 + 11 with HOH	Gly50 Lys67 HOH 596 HOH 336 HOH 352 HOH 412 HOH 462 HOH 543 HOH 338 HOH 459 HOH 462 HOH 459 HOH 543	H-accept H-accept H-donor H-accept H-accept H-accept H-accept H-accept H-accept H-accept H-accept H-accept H-accept	3.17 2.96 3.15 3.48 2.52 2.54 2.52 3.27 2.75 2.58 2.95 2.82 3.00

**Fig. 2:**
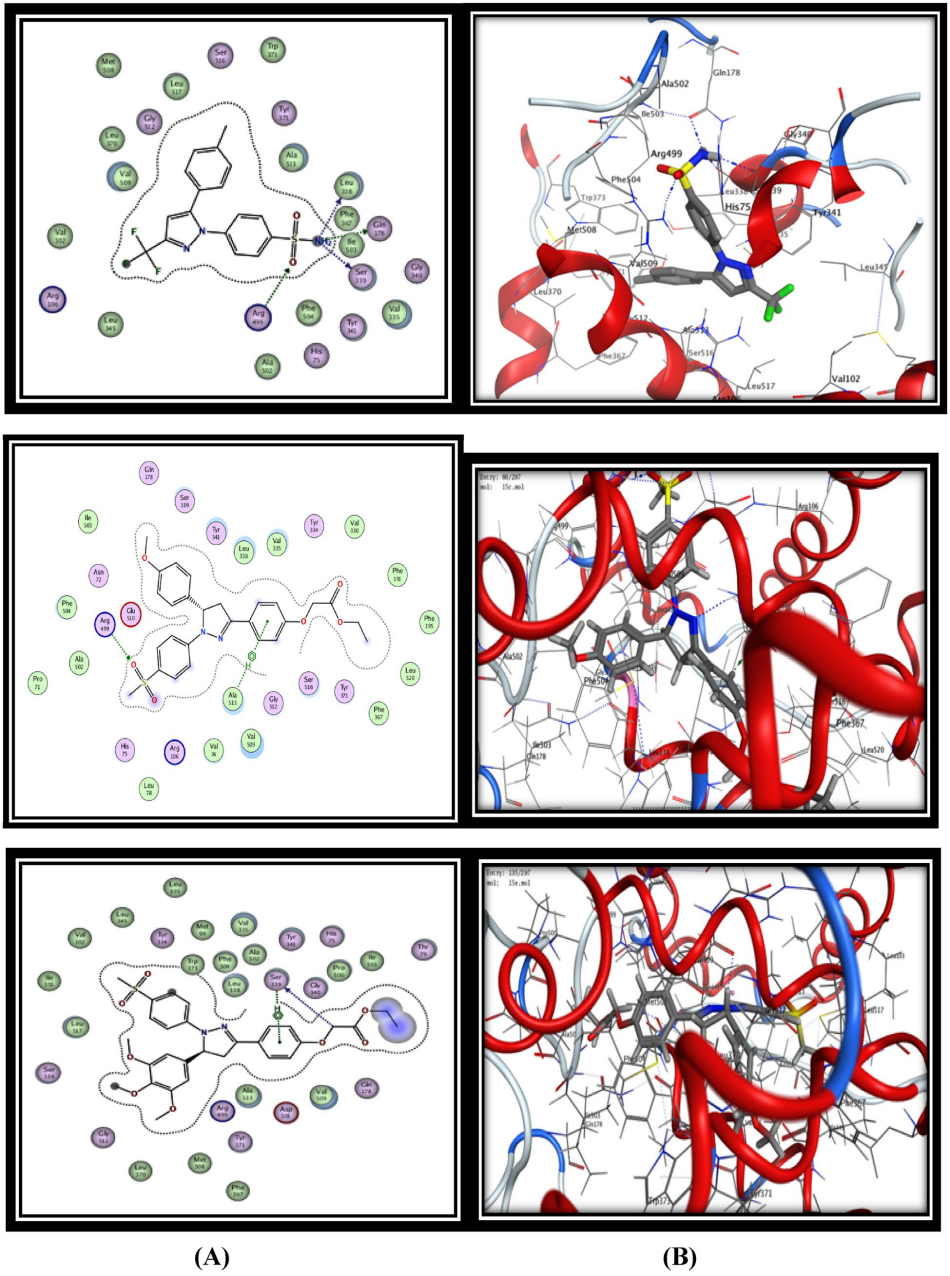

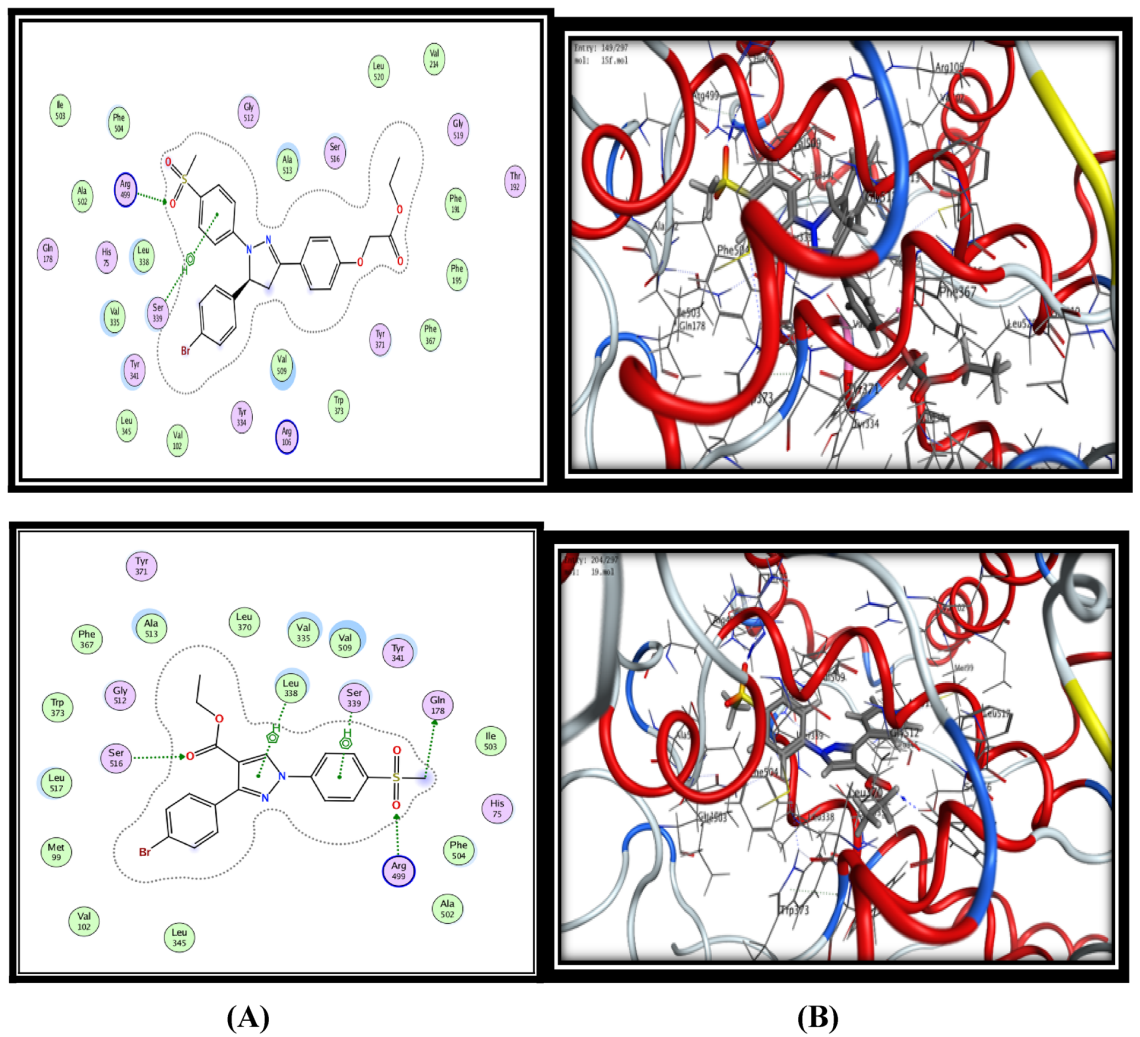
Binding of ligand celecoxib, compound **15c**, **15d, 15 h** and** 19d** inside COX-2 active site. **A** 2D interactions of the proposed binding mode of celecoxib or selected compound inside the active site of COX-2. **B** 3D interactions of celecoxib or selected compond

Regarding COX-2 enzyme, docking of celecoxib into the COX-2 isozyme afforded four hydrogen bonding interactions (HBs) and (distance Aº) **(i)** SO_2_NH_2_ with Leu338 (3.04 Aº), **(ii)** SO_2_NH_2_ with Ser339 (2.93 Aº), **(iii)** SO_2_NH_2_ with Gln178 (3.06 Aº), **(iv)** SO_2_NH_2_ with Arg499 (3.53 Aº) with energy score -13.57 kcal/mol (Fig. 2). The docking results of the synthesized compounds fitted well to COX-2 active site inside the pocket and showed variable binding mode of interactions with energy score ranged from −12.90 to −18.17 kcal/mol. The binding mode of interactions for the most active compounds **15c, 15d, 15 h** and** 19d** with COX-2 active site were represented as in Fig. 2. They exhibited higher binding affinity toward COX-2 enzyme by forming 2–5 hydrogen-bond interactions in addition to arene-hydrogen bonds with different amino acids such as (Leu338, Ser339, Gln178, and Arg449) similar to the ligand compound in addition to Ser516 and Ala513 amino acids with good energy scores range (−17.64 to −18.17 kcal/mole) and these results elucidate their good selectivity toward COX-2.

Concerning the docking results of the most active compound **19d**, it was observed that its binding mode in COX-2 active site was very similar to celecoxib mode, it showed three hydrogen bonding interaction (HBs) **(i)** benzene ring with Ser339 (3.99 Aº), **(ii)** SO_2_CH_3_ with Gln178 (3.46 Aº), **(iii)** COOC_2_H_5_ with Ser516 (3.00 Aº), in addition to two hydrophobic π-H interactions with the key amino **(iv)** SO_2_CH_3_ with Arg499 (2.71 Aº), and **(v)** pyrazole ring with Leu338 (4.21 Aº) with energy score -18.17 kcal/mol (Fig. 2). On the other hand, the binding mode of the least active selective COX-2 compound **15a** showed only one hydrogen bond interaction with Ser339 (4.00Aº) with energy score -12.90 kcal/mol. The docking results including energy scores, hydrogen bonding interaction between amino acid residues and functional groups of docked compounds and their length were summarized in Table 6.

**Fig. 3:**
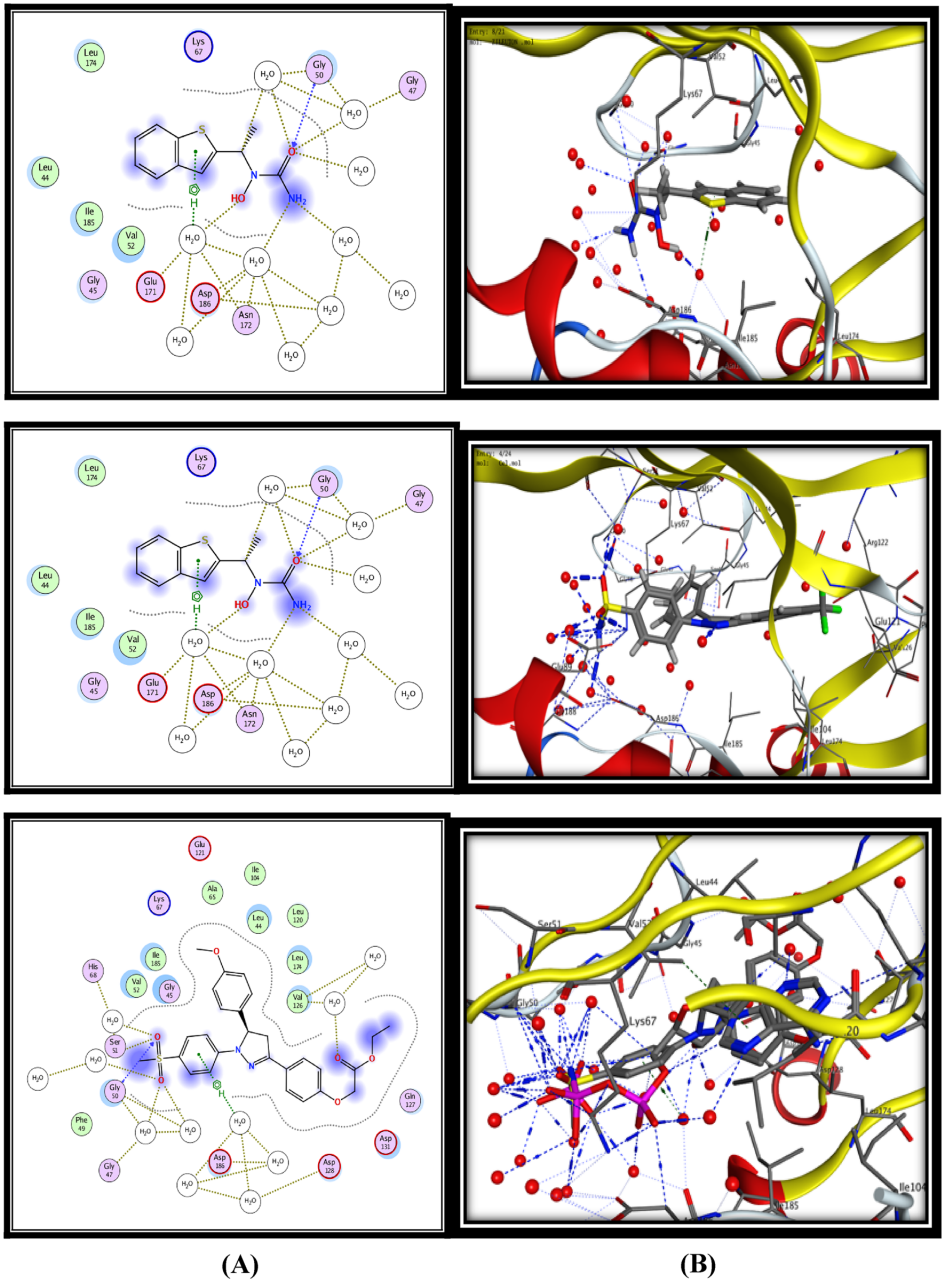

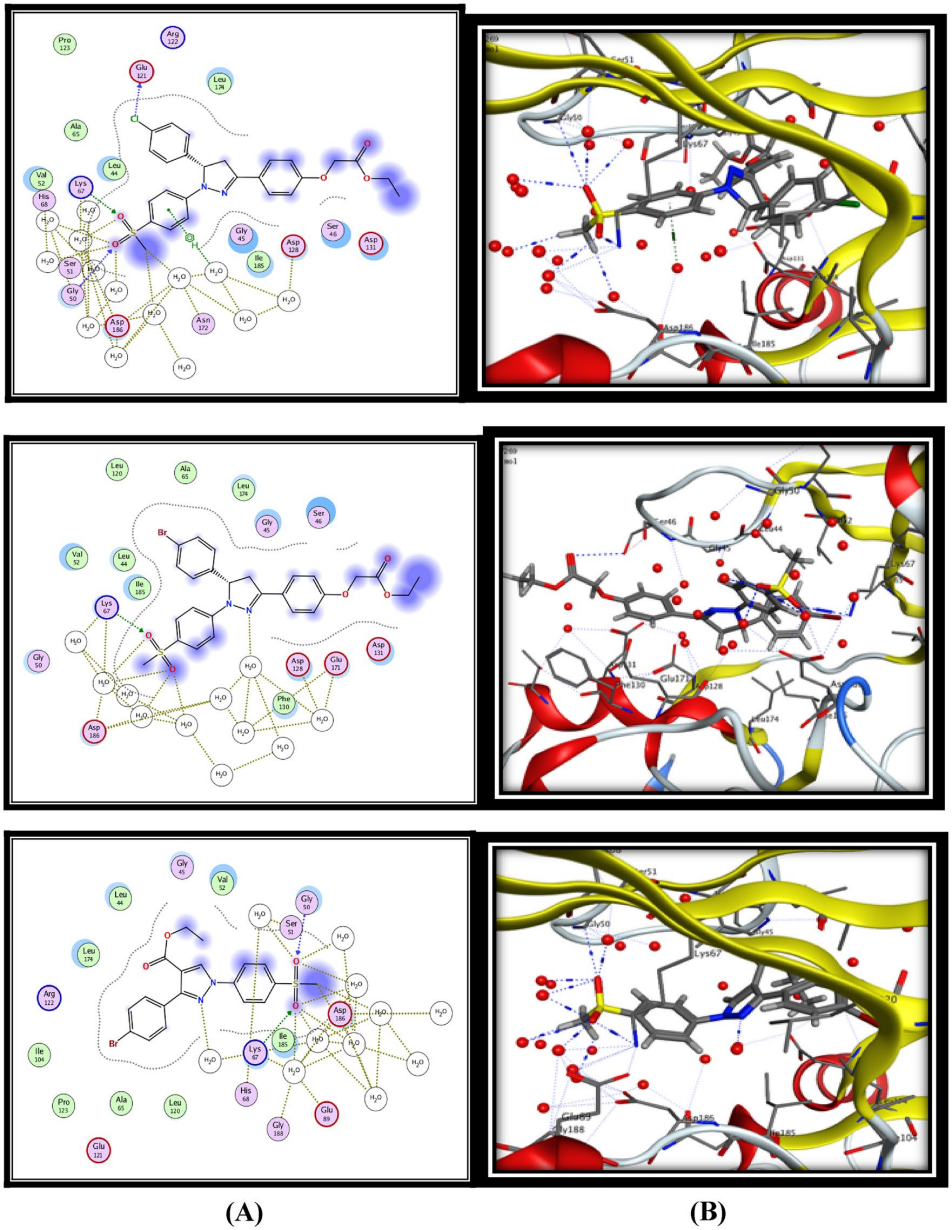
Binding of zileuton, compound **15c**, **15d, 15 h** and** 19d** inside 5-LOX active site. **A** 2D interactions of the proposed binding mode of zileuton or selected compound inside the active site of COX-2. **B** 3D interactions of zileuton or selected compound

For 5-LOX enzyme, the binding pattern of zileuton revealed a H-bond interaction between linker (O) and the key amino acid Gly50 (3.60 Aº), in addition to eight hydrogen bonds and hydrophobic π−H interactions with water molecules with energy score −12.00 kcal/mol (Fig. 3). On the other hand celecoxib showed two H-bond interactions between (O) of (SO_2_NH_2_) and the key amino acid Gly50 and Lys67 (3.17 Aº) and (2.96 Aº) respectively, in addition to eleven hydrogen bonds with water molecules with energy score −13.49 kcal/mol (Fig. 3). The results of the present in silico docking simulation for the most active compounds **15c, 15d, 15 h** and** 19d** with 5-LOX active site revealed that they showed good binding interaction by making at least one hydrogen bond interaction with the main amino acids, in addition to several hydrogen bonds and hydrophobic π−H interactions with water molecules in similar manner to the both ligands (zileuton and celecoxib) with energy score range (−1.78 to −18.92 kcal/mol) (Fig. 3).

The docking results of the significant compound **19d** showed two hydrogen bonding interactions (HBs) **(i)** SO_2_CH_3_with Gly50 (3.05 Aº), **(ii)** SO_2_CH_3_ with Lys67 (3.09 Aº), in addition to ten hydrogen bonds with water molecules with low energy score -18.92 kcal/mol (Fig. 3). While the docking results of compound **15 h** which showed the lower 5-LOX inhibitory activity exhibited that, it form only one H-bond with Lys67 amino acid (2.83 Aº) with high energy score (−11.78 kcal/mol), in addition to formation of the least number of hydrogen bonds with water molecules (5 H-bonds) and that illustrate its lower 5-LOX inhibitory activity (Fig. 3).

The docking results including energy scores, hydrogen bonding interaction between amino acid residues and functional groups of docked compounds and their length were represented in Table 7.

The results confirmed that there is a parallel relationship between docking study and the in vivo anti-inflammatory activity.

## SwissADME studies

Drug development involves the assessment of efficacy and toxicity of the new drug candidates. A critical piece in drug discovery and development is conducting DMPK (drug metabolism and pharmacokinetics) studies, often referred to as ADMET (absorption, distribution, metabolism, elimination and toxicity) studies. The initiation of early absorption, distribution, metabolism and excretion (ADME) screening has dramatically decreased the proportion of compounds failing in clinical trials [54].

ADME study was assessed to new most active synthesized compounds **15c, 15d, 15 h**,** 19d**, reference drugs celecoxib and lonazolac using the SwissADME web tool. Many parameters were detected, which are presented in (Table 8).

**Table 8:** Physicochemical, Pharmacokinetics parameters, lipophilicity, water solubility and drug likeness of compounds **15c, 15d, 15 h, 19d** and celecxoib

Compound No	M.W	No of rotoable atoms	No of H-bond acceptors	No of H-bond donors	Molar refractivity	TPSA (Å^2^)	Log (p_0/w_)	Log S (ESOl)	GI absorption	BBB permeant	CYP1A2 inhibitor	CYP2C19 inhibitor	CYP2C9 inhibitor	Lipinski	Bioavailability score	Lead likeness	Pains
15c	508.59	10	7	0	144.05	102.88	3.95	−5.53	High	No	No	Yes	Yes	1 violation	0.55	3 violations	0 alert
15d	568.64	12	9	0	157.03	121.34	3.78	−5.70	High	No	No	Yes	Yes	1 violation	0.55	3 violations	0 alert
14 h	557.46	9	6	0	145.25	93.65	4.03	−6.37	High	No	No	Yes	Yes	1 violation	0.55	3 violations	0 alert
19d	449.32	6	5	0	105.88	86.64	3.43	−5.03	High	No	Yes	Yes	Yes	0 violations	0.55	2 violations	0 alert
Celecoxib	381.34	4	7	1	89.96	86.36	2.56	−4.57	High	No	Yes	No	Yes	0 violations	0.55	1 violation	0 alert
Lonazolac	312.75	4	3	1	85.55	55.12	2.60	−4.49	High	Yes	No	Yes	Yes	0 violations	0.85	1 violation	0 alert

**Fig. 4:**
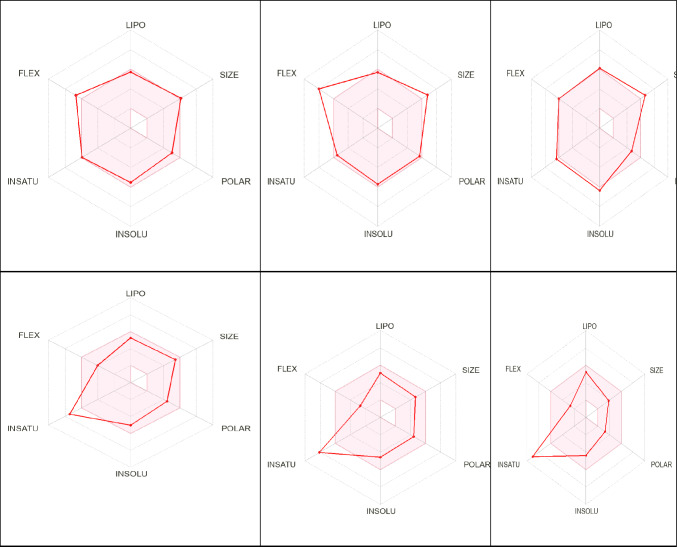
Physicochemical diagram of the most active compounds **15c**, **15d**, **15 h**, **19d** and celecoxib

Drug likeliness parameters were studied by using different rules such as Lipinski rule of five, Ghose rule, Veber, Egan rule, and Muegge rule [54]. Particularly, the compliance of compound to taraditional Lipiniskiʼs rule “rule of five” which indicates that orally active substance in humans is an important indication for drugs pharmacokinetics. This simple rule states that oral active drug has no more than one violation of the following criteria: molecular weight (M wt) less than 500 Dalton (Da); no more than five hydrogen bond donors (HBD); no more than 10 hydrogen bond acceptors (HBA); and calculated octanol–water partition coefficient (clogP) not greater than 5 or (MlogP) not greater than 4.15 [55]. Also, topological polar surface area (TPSA) and the number of routable bonds are other critical properties that have been linked to the drug bioavailability. The reports suggested that compounds with a TPSA of more than 140 Å^2^ and more than ten routable bonds are thought to have low oral bioavailability (Fig. 4) [55].

From the obtained results, compounds **15c, 15d,** and** 15 h** have M wt higher than 500 gm/ mol ranged from 508.59 to 568.64. Also, they have number of routable bonds (9 to 12) in comparison with number of routable bonds of standard drugs celecoxib and lonazolac. Furthermore, compounds **15c, 15d,** and** 15 h** have no H-bond donors in comparison with celecoxib. In addition, **15c, 15d,** and** 15 h** compounds have number of H-bond acceptors (6 to 9) of 1 relative to celecoxib and lonazolac (Table 8**)**. The TPSA of compounds **15c, 15d** and** 15 h** are ranged from 93.65 to 121.34 Å^2^ within standard range 140 Å^2^ in comparison with that of celecoxib (86.36 Å^2^) and lonazolac (55.12 Å^2^). Sufficient lipophilicity is required for having a good biological activity; their parameters can be determined by using MLOGP [56]. Concerning lipophilicity all compounds displayed the least value of partition coefficient 3.78–4.03 (Log P ≤ 5) relative to that of 2.56 of celecoxib (Table 8).

Fortunately, the most active compound (**19d**) fulfilled all Lipinskisʼ guidelines similar to the clinically used celecoxib and lonazolac, including molecular weight 449.3 Da compared to the molecular weight of celecoxib (381.76 Da) and that of lonazolac (312.75 Da). The number of hydrogen bond donors is zero compared to one HBD in celecoxib**.** Hydrogen bond acceptors were 5 compared to seven HBA in celecoxib and lonazolac (Table 8). The TPSA of compound (**19d**) is within standard range 140 Å^2^ equal to 86.64 Å^2^ which was very similar to of celecoxib (86.36 Å^2^) and lonazolac (55.12 Å^2^). Additionally, the partition coefficient of it was 3.43 relative to that of 2.56 of celecoxib and 2.60 for lonazolac (Table 8).

As our synthesized compounds have TPSA larger than 60 Å, all compounds don’t pass blood brain barrier as observed by our calculations. The bioavailability of drugs is affected by gastrointestinal absorption. All tested compounds have high GIT absorption comparable to celecoxib and lonazolac.

Cytochromes P450 (CYPs) are a family of enzymes that are responsible for over 90% of oxidative metabolic processes and play a major role in breaking down a variety of endogenous and xenobiotic substances [54–56]. Inhibition of CYP enzymes causes failure in inhibitory drug metabolism. As a result, studying the inhibitory activity of proposed derivatives against a certain CYP is very important in medication development. The results of the inhibitory prediction for two CYP isoforms (CYP1A2, and CYP2C19) are showed in (Table 8). All our evaluated compounds were anticipated to inhibit both CYP2C9 enzymes, which means they are lower exposed to inhibitory drug metabolism.

Regarding to bioavailability score, all compounds had similar score to that of reference drugs celecoxib (0.55) and lonazolac (0.85) which means that all ligands can reach the systemic circulation (Table 8).

In conclusion, the ADME studies support the discovery of the prepared compounds **15c, 15d, 15 h** and** 19d** as active oral bio-available anti-inflammatory drugs.

### Structural activity relationship (SAR)

**Fig. 5:**
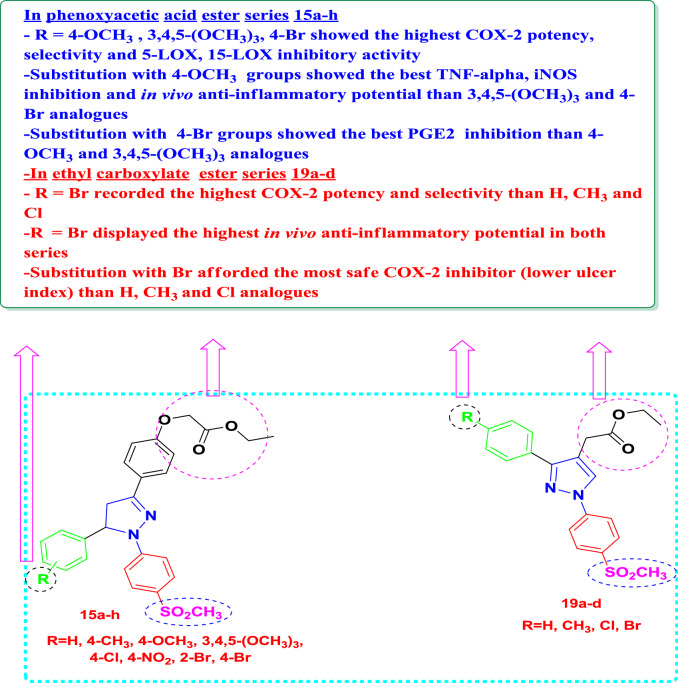
SAR study of the target pyrazole ester derivatives **15a-h** and** 19a-d** as anti-inflammatory agents

SAR was performed based on the biological activity results of di-aryl/tri-aryl substituted pyrazole ester derivatives **15a-h** and** 19a-d** as anti-inflammatory agents, as shown in Fig. 5. It was evident that in phenoxyacetic acid ester series **15a-h** when R = 4-OCH_3_, 3,4,5-(OCH_3_)_3_, 4-Br, these derivatives (**15c, 15d** and** 15 h**) showed the highest COX-2 potency, selectivity and 5-LOX, 15-LOX inhibitory activity. In addition, substitution with 4-OCH_3_ group as in compound **15c** showed the best TNF-α, iNOS inhibition and in vivo anti-inflammatory potential than 3,4,5-(OCH_3_)_3_ and 4-Br analogues. Moreover, substitution with 4-Br group as in compound **15 h** exhibited the best PGE2 inhibition than 4-OCH_3_ and 3,4,5-(OCH_3_)_3_ analogues.

On the other hand, in ethyl carboxylate ester series **19a-d** when R = Br this derivative **19d** recorded the highest COX-2 potency and selectivity than H, CH_3_ and Cl. Furthermore, when R = Br the same derivative displayed the highest in vivo anti-inflammatory potential in both series. Substitution with Br afforded the safest COX-2 inhibitor (lower ulcer index) than H, CH_3_ and Cl analogues.

### Modifications in anti-inflammatory activity upon replacement of substituents or rings from reported compounds

- Incorporation of **SO**_**2**_**CH**_**3**_** pharmacophore** in the target compounds results in increase of COX-2 selectivity comparable to lonazolac.
- Replacement of **4-chlorophenyl** moiety at pyrazole C-3 of lonazolac with electron-donating groups like 4-methoxyphenyl and 3,4,5-trimethoxyphenyl in the target compounds **15a-h** and **19a-d** increased COX-2 selectivity (COX-2 S.I. **28.56 and 70.22**, respectively).
- Replacement of** acetic acid** moiety in position-4 of the pyrazole nucleus of lonazolac **(3)** or the hydrazone moity of reported compounds **4a-c** (COX-2 S.I. = 9.22–98.7) with steric arm **(phenoxyacetic acid ester)** as in target compounds **15a-h** or **(ethyl carboxylate moiety)** as in target derivatives **19a-d**, increased COX-2 selectivity (COX-2 S.I. 28.56 and 70.22, respectively).
- Keeping **Y-shaped diaryl pyrazole scaffold** in the target compounds resembling to the previously reported excellent COX-2 selective inhibitors **(7a-c** COX-2 S.I. **50.6–253.1)** resulted in keeping the high COX-2 selectivity to some extent.
- Replacement of **hydrazone moiety** in position 4 of pyrazole ring of the reported compounds **8a**, **8b**, **9a** and **9b** increased COX-2 selectivity from **(8.41–10.55) to (28.56–70.22).** In addition of increasing 5-LOX potency from **(1.92, 2.31 µM** compounds **8a**, **8b**) to **(0.241–0.81 µM** of our target compounds).
- Replacement of **acid moiety** of compound **10** with ester scaffold increased 5-LOX potency from 5.88 µM to (0.241–0.81 µM of our target compounds).

## Experimental

### Chemistry

Melting points were determined on a Griffin apparatus and are uncorrected. Infrared (IR) spectra were recorded on a Shimadzu 435 spectrometer using KBr discs. ^1^H NMR and ^13^C NMR spectra were measured on a Bruker 400 MHz spectrometer (Faculty of Pharmacy, Beni-Suef University, Beni-Suef, Egypt) in D_2_O, (DMSO-d_6_) with TMS as the internal standard, where *J* (coupling constant) values were estimated in hertz (*Hz*). Mass spectra were run on Hewlett Packard 5988 spectrometer. Microanalysis was performed for C, H, N at the Micro Analytical Center, Cairo University, Egypt and was within ± 0.4% of theoretical values. All other reagents, purchased from the Acros Chemical Company (Milwaukee, WI). The intermediates 4-methanesulfonylphenylhydrazine hydrochloride **12** [47, 48], chalcone derivatives **13a-h** [44–46]**,** hydrazone derivatives** 17a-d** and the pyrazole aldehydes** 18a-d** [19] were prepared according to reported procedure.

### General method for preparation of 1,3,5-triarylpyrazolines (15a-h)

A solution of the appropriate chalcone (**13a-h**, 0.1 mol) in ethanol (50 mL) was heated under reflux with 4-methanesulfonylphenylhydrazine hydrochloride (**12,** 0.1 mol) for 36 h, cooled and diluted with cold water. The precipitated crude product was filtered and recrystallized from ethanol to give **15a-h**. Physical and spectral data are listed below:

#### Ethyl 2-(4-(1-(4-(methylsulfonyl)phenyl)-5-phenyl-4,5-dihydro-1*H*-pyrazol-3-yl)phenoxy) acetate (15a)

79% yield; reddish brown solid; m.p. 232–234 °C; IR (KBr disk) 1732 (C = O), 1377, 1138 (SO_2_); ^1^H NMR (DMSO-d_6_) δ 1.23 (t, *J* = 3.2 Hz, 2H, OCH_2_CH_3_), 3.07 (s, 3H, SO_2_CH_3_), 3.15 (dd, *J* = 5.2, 17.6 Hz, 1H, pyrazole H-4), 3.93 (dd, *J* = 12.0, 17.6 Hz, 1H, pyrazole H'-4), 4.15 (q, *J* = 3.2 Hz, 3H, OCH_2_CH_3_), 4.85 (s, 2H, CH_2_), 5.59 (dd, *J* = 4.8, 12.0 Hz, 1H, pyrazole H-5), 7.03 (d, *J* = 8.8 Hz, 2H, phenyl H-2, H-6), 7.09 (d, *J* = 8.8 Hz, 2H, phenoxy H-3, H-5), 7.26 (m, 3H, phenyl H-3, H-4, H-5), 7.34 (d, *J* = 7.2 Hz, 2H, aminosulfonylphenyl H-3, H-5), 7.64 (d, *J* = 8.4 Hz, 2H, phenoxy H-2, H-6), 7.74 (d, *J* = 8.4 Hz, 2H, aminosulfonlphenyl H-2, H-6); ^13^C NMR (DMSO-d_6_) δ 14.52, 43.75, 44.66, 61.20, 62.66, 65.19, 112.43, 115.30, 125.31, 126.17, 128.30, 129.02, 129.19, 129.65, 142.09, 147.66, 150.84, 159.21, 168.97; MS (m/z, relative abundance %): 478.16 (M^+.^); Anal. Calcd for C_26_H_26_N_2_O_5_S: C, 65.25; H, 5.48; N, 5.85; Found: C, 65.44; H, 5.32; N, 5.55.

#### Ethyl 2-(4-(1-(4-(methylsulfonyl)phenyl)-5-(*p*-tolyl)-4,5-dihydro-1*H*-pyrazol-3-yl)phenoxy)acetate (15b)

75% yield; yellowish brown solid; m.p. 244–246 °C; IR (KBr disk) 1743 (C = O), 1334, 1141 (SO_2_); ^1^H NMR (DMSO-d_6_) δ 1.20 (t, *J* = 7.2 Hz, 2H, OCH_2_CH_3_), 3.71 (s, 3H, CH_3_), 3.06 (s, 3H, SO_2_CH_3_), 3.11 (dd, *J* = 4.8, 17.6 Hz, 1H, pyrazole H-4), 3.90 (dd, *J* = 12.0, 17.2 Hz, 1H, pyrazole H'-4), 4.15 (q, *J* = 7.2 Hz, 3H, OCH_2_CH_3_), 4.84 (s, 2H, CH_2_), 5.54 (dd, *J* = 4.8, 12.0 Hz, 1H, pyrazole H-5), 7.00 (d, *J* = 8.4 Hz, 2H, 4-methylphenyl H-3, H-5), 7.08 (d, *J* = 8.8 Hz, 2H, phenoxy H-3, H-5), 7.15 (m, 4H, 4-methylphenyl H-2, H-6, aminosulfonylphenyl H-3, H-5), 7.62 (d, *J* = 8.8 Hz, 2H, phenoxy H-2, H-6), 7.72 (d, *J* = 8.8 Hz, 2H, aminosulfonlphenyl H-2, H-6); ^13^C NMR (DMSO-d_6_) δ 14.49, 21.07, 43.90, 44.64, 62.36, 62.43, 65.15, 112.44, 115.29, 125.34, 126.09, 128.27, 128.97, 130.18, 137.42, 139.05, 147.65, 150.84, 159.16, 169.01; MS (m/z, relative abundance %): 531.17 [M + K]^**+**^; Anal. Calcd for C_27_H_28_N_2_O_5_S: C, 65.83; H, 5.73; N, 5.69; Found: C, 65.74; H, 5.78; N, 5.45.

#### Ethyl 2-(4-(5-(4-methoxyphenyl)-1-(4-(methylsulfonyl)phenyl)-4,5-dihydro-1*H*-pyrazol-3-yl)phenoxy)acetate (15c)

83% yield; yellowish brown solid; m.p. 222–224 °C; IR (KBr disk) 1743 (C = O), 1334, 1141 (SO_2_); ^1^H NMR (DMSO-d_6_) δ 1.21 (t, *J* = 7.2 Hz, 2H, OCH_2_CH_3_), 3.07 (s, 3H, SO_2_CH_3_), 3.12 (dd, *J* = 4.8, 17.6 Hz, 1H, pyrazole H-4), 3.71 (s, 3H, OCH_3_), 3.89 (dd, *J* = 12.0, 17.6 Hz, 1H, pyrazole H'-4), 4.15 (q, *J* = 7.2 Hz, 3H, OCH_2_CH_3_), 4.85 (s, 2H, CH_2_), 5.54 (dd, *J* = 4.8, 12.0 Hz, 1H, pyrazole H-5), 6.89 (d, *J* = 8.4 Hz, 2H, 4-methoxyphenyl H-3, H-5), 7.01 (d, *J* = 8.8 Hz, 2H, phenoxy H-3, H-5), 7.10 (d, *J* = 8.8 Hz, 2H, 4-methoxyphenyl H-2, H-6), 7.18 (d, *J* = 8.8 Hz, 2H, aminosulfonylphenyl H-3, H-5), 7.63 (d, *J* = 8.8 Hz, 2H, phenoxy H-2, H-6), 7.73 (d, *J* = 8.4 Hz, 2H, aminosulfonlphenyl H-2, H-6); ^13^C NMR (DMSO-d_6_) δ 14.52, 43.75, 44.67, 55.53, 61.20, 62.17, 65.19, 112.45, 114.98, 115.30, 125.40, 127.45, 128.26, 128.97, 129.07, 133.96, 147.65, 150.84, 159.13, 159.18, 168.98; MS (m/z, relative abundance %): 508.17 (M^+.^); Anal. Calcd for C_27_H_28_N_2_O_6_S: C, 63.76; H, 5.55; N, 5.51; Found: C, 63.64; H, 5.68; N, 5.25.

#### Ethyl 2-(4-(1-(4-(methylsulfonyl)phenyl)-5-(3,4,5-trimethoxyphenyl)-4,5-dihydro-1*H*-pyrazol-3-yl)phenoxy)acetate (15d)

88% yield; white solid; m.p. 256–258 °C; IR (KBr disk) 1751 (C = O), 1323, 1130 (SO_2_); ^1^H NMR (DMSO-d_6_) δ 1.20 (t, *J* = 7.2 Hz, 2H, OCH_2_CH_3_), 3.08 (s, 3H, SO_2_CH_3_), 3.12 (dd, *J* = 4.8, 17.6 Hz, 1H, pyrazole H-4), 3.63 (s, 3H, 4-methoxyphenyl), 3.70 (s, 6 H, 3,5-dimethoxyphenyl), 3.91 (dd, *J* = 12.0, 17.6 Hz, 1H, pyrazole H'-4), 4.15 (q, *J* = 7.2 Hz, 3H, OCH_2_CH_3_), 4.85 (s, 2H, CH_2_), 5.45 (dd, *J* = 4.8, 12.0 Hz, 1H, pyrazole H-5), 6.59 (s, 2H, 3,4,5-trimethoxyphenyl H-2, H-6), 7.01 (d, *J* = 8.8 Hz, 2H, phenoxy H-3, H-5), 7.13 (d, *J* = 8.8 Hz, 2H, aminosulfonylphenyl H-3, H-5), 7.67 (d, *J* = 8.8 Hz, 2H, phenoxy H-2, H-6), 7.74 (d, *J* = 8.8 Hz, 2H, aminosulfonlphenyl H-2, H-6); ^13^C NMR (DMSO-d_6_) δ 14.51, 43.85, 44.66, 56.37, 56.42, 61.20, 63.30, 65.00, 65.17, 103.33, 112.58, 115.28, 125.12, 125.33, 128.32, 129.00, 129.35, 137.24, 137.96, 148.14, 151.03, 153.87, 159.20, 169.00, 170.42; MS (m/z, relative abundance %): 591.19 [M + Na]^**+**^; Anal. Calcd for C_29_H_32_N_2_O_8_S: C, 61.25; H, 5.67; N, 4.93; Found: C, 61.44; H, 5.88; N, 5.15.

#### Ethyl 2-(4-(5-(4-chlorophenyl)-1-(4-(methylsulfonyl)phenyl)-4,5-dihydro-1*H*-pyrazol-3-yl)phenoxy)acetate (15e)

69% yield; yellowish white solid; m.p. 218–220 °C; IR (KBr disk) 1755 (C = O), 1334, 1141 (SO_2_); ^1^H NMR (DMSO-d_6_) δ 1.20 (t, *J* = 6.8 Hz, 2H, OCH_2_CH_3_), 3.07 (s, 3H, SO_2_CH_3_), 3.15 (dd, *J* = 4.8, 17.6 Hz, 1H, pyrazole H-4), 3.92 (dd, *J* = 12.0, 17.6 Hz, 1H, pyrazole H'-4), 4.15 (q, *J* = 6.8 Hz, 3H, OCH_2_CH_3_), 4.84 (s, 2H, CH_2_), 5.62 (dd, *J* = 5.2, 12.4 Hz, 1H, pyrazole H-5), 7.00 (d, *J* = 8.8 Hz, 2H, 4-chlorophenyl H-3, H-5), 7.08 (d, *J* = 8.8 Hz, 2H, phenoxy H-3, H-5), 7.27 (d, *J* = 8.8 Hz, 2H, 4-chlorophenyl H-2, H-6), 7.40 (d, *J* = 8.4 Hz, 2H, aminosulfonylphenyl H-3, H-5), 7.67 (d, *J* = 8.8 Hz, 2H, phenoxy H-2, H-6), 7.73 (d, *J* = 8.8 Hz, 2H, aminosulfonlphenyl H-2, H-6); ^13^C NMR (DMSO-d_6_) δ 14.51, 43.52, 44.65, 61.17, 61.95, 62.18, 65.19, 112.47, 115.30, 125.20, 128.19, 128.33, 129.08, 129.24, 129.63, 132.69, 140.99, 147.50, 150.88, 159.25, 168.97; MS (m/z, relative abundance %): 512.12 (M +); Anal. Calcd for C_26_H_25_ClN_2_O_5_S: C, 60.87; H, 4.91; N, 5.46; Found: C, 60.78; H, 4.82; N, 5.65.

#### Ethyl 2-(4-(1-(4-(methylsulfonyl) phenyl)-5-(4-nitrophenyl)-4,5-dihydro-1*H*-pyrazol-3-yl)phenoxy)acetate (15f)

61% yield; yellowish white solid; m.p. 238–240 °C; IR (KBr disk) 1751 (C = O), 1343, 1141 (SO_2_); ^1^H NMR (DMSO-d_6_) δ 1.20 (t, *J* = 6.8 Hz, 2H, OCH_2_CH_3_), 3.08 (s, 3H, SO_2_CH_3_), 3.22 (dd, *J* = 4.8, 17.6 Hz, 1H, pyrazole H-4), 3.99 (dd, *J* = 12.4, 17.6 Hz, 1H, pyrazole H'-4), 4.15 (q, *J* = 6.8 Hz, 3H, OCH_2_CH_3_), 4.85 (s, 2H, CH_2_), 5.81 (dd, *J* = 4.8, 12.0 Hz, 1H, pyrazole H-5), 7.01 (d, *J* = 8.8 Hz, 2H, 4-nitrophenyl H-3, H-5), 7.09 (d, *J* = 8.8 Hz, 2H, phenoxy H-3, H-5), 7.54 (d, *J* = 8.4 Hz, 2H, 4-nitrophenyl H-2, H-6), 7.66 (d, *J* = 8.4 Hz, 2H, aminosulfonylphenyl H-3, H-5), 7.74 (d, *J* = 8.8 Hz, 2H, phenoxy H-2, H-6), 8.22 (d, *J* = 8.8 Hz, 2H, aminosulfonlphenyl H-2, H-6); ^13^C NMR (DMSO-d_6_) δ 14.52, 44.63, 44.69, 61.99, 65.19, 112.52, 115.32, 124.92, 125.04, 127.75, 128.42, 129.17, 129.70, 147.41, 147.51, 149.50, 150.97, 159.34, 168.95; MS (m/z, relative abundance %): 523.14 (M +); Anal. Calcd for C_26_H_25_N_3_O_7_S: C, 59.65; H, 4.81; N, 8.03; Found: C, 59.86; H, 4.98; N, 8.16.

#### Ethyl 2-(4-(5-(2-bromophenyl)-1-(4-(methylsulfonyl)phenyl)-4,5-dihydro-1*H*-pyrazol-3-yl)phenoxy)acetate (15 g)

68% yield; yellowish white solid; m.p. 278–280 °C; IR (KBr disk) 1735 (C = O), 1300, 1141 (SO_2_); ^1^H NMR (DMSO-d_6_) δ 1.20 (t, *J* = 7.2 Hz, 2H, OCH_2_CH_3_), 3.08 (s, 3H, SO_2_CH_3_), 3.16 (dd, *J* = 5.2, 17.6 Hz, 1H, pyrazole H-4), 4.03 (dd, *J* = 12.0, 17.6 Hz, 1H, pyrazole H'-4), 4.15 (q, *J* = 7.2 Hz, 3H, OCH_2_CH_3_), 4.84 (s, 2H, CH_2_), 5.72 (dd, *J* = 5.2, 12.0 Hz, 1H, pyrazole H-5), 6.96 (d, *J* = 8.8 Hz, 2H, phenoxy H-3, H-5), 7.00 (d, *J* = 2.4 Hz, 2H, 2-bromophenyl H-6), 7.02 (d, *J* = 8.8 Hz, 2H, phenoxy H-2, H-6), 7.25 (t, *J* = 6.0 Hz, 2H, 2-bromophenyl H-5), 7.31 (t, *J* = 7.6 Hz, 2H, 2-bromophenyl H-4), 7.68 (d, *J* = 8.4 Hz, 2H, aminosulfonylphenyl H-3, H-5), 7.75 (d, *J* = 2.0 Hz, 2H, 2-bromophenyl H-6), 7.76 (d, *J* = 8.8 Hz, 2H, aminosulfonlphenyl H-2, H-6); ^13^C NMR (DMSO-d_6_) δ 14.51, 42.53, 44.64, 61.21, 62.63, 65.18, 112.27, 115.29, 121.80, 125.08, 128.28, 128.39, 129.14, 129.23, 129.57, 130.34, 133.90, 139.87, 147.32, 150.97, 159.28, 168.97; MS (m/z, relative abundance %): 595.07 [M + K]^**+**^; Anal. Calcd for C_26_H_25_BrN_2_O_5_S: C, 56.02; H, 4.52; N, 5.03; Found: C, 56.08; H, 4.58; N, 5.16.

#### Ethyl 2-(4-(5-(4-bromophenyl)-1-(4-(methylsulfonyl)phenyl)-4,5-dihydro-1*H*-pyrazol-3-yl)phenoxy)acetate (15 h)

65% yield; yellowish white solid; m.p. 232–234 °C; IR (KBr disk) 1739 (C = O), 1400, 1141 (SO_2_); ^1^H NMR (DMSO-d_6_) δ 1.20 (t, *J* = 6.8 Hz, 2H, OCH_2_CH_3_), 3.07 (s, 3H, SO_2_CH_3_), 3.17 (dd, *J* = 4.8, 17.6 Hz, 1H, pyrazole H-4), 3.92 (dd, *J* = 12.0, 17.6 Hz, 1H, pyrazole H'-4), 4.15 (q, *J* = 6.8 Hz, 3H, OCH_2_CH_3_), 4.85 (s, 2H, CH_2_), 5.62 (dd, *J* = 4.8, 12.0 Hz, 1H, pyrazole H-5), 7.01 (d, *J* = 8.8 Hz, 2H, phenoxy H-3, H-5), 7.09 (d, *J* = 8.8 Hz, 2H, 4-bromophenyl H-3, H-5), 7.22 (d, *J* = 8.4 Hz, 2H, phenoxy H-2, H-6), 7.54 (d, *J* = 8.8 Hz, 2H, 4-bromophenyl H-2, H-6), 7.65 (d, *J* = 8.8 Hz, 2H, aminosulfonylphenyl H-3, H-5), 7.73 (d, *J* = 8.8 Hz, 2H, aminosulfonlphenyl H-2, H-6); ^13^C NMR (DMSO-d_6_) δ 14.52, 43.47, 44.66, 61.20, 65.19, 112.46, 115.31, 121.21, 125.20, 128.34, 128.55, 129.09, 129.40, 132.55, 141.43, 147.48, 150.90, 159.26, 168.97; MS (m/z, relative abundance %): 595.07 [M + K]^**+**^; Anal. Calcd for C_26_H_25_BrN_2_O_5_S: C, 56.02; H, 4.52; N, 5.03; Found: C, 56.25; H, 4.80; N, 5.40.

### General procedure for synthesis of the pyrazolyl ester derivatives (19a-d)

A mixture of the appropriate pyrazole aldehyde (**18a-d**, 1.0 mmol) and oxone (0.92 g, 1.5 mmol) in ethanol (20 mL) was stirred at room temperature for 24 h. After completion of the reaction the solvent was evaporated, and the formed precipitate was washed with water (150 mL), the obtained residue was recrystallized from methanol to give the final pyrazolyl ester derivatives **(19a-d)**: Physical and spectral data are listed below:

#### Ethyl 1-(4-(methylsulfonyl)phenyl)-3-phenyl-1*H*-pyrazole-5-carboxylate (19a)

Yield 75%; white solid; m.p. 256–258 °C; IR (KBr disk) 1701 (C = O), 1355, 1149 (SO_2_); ^1^H NMR (DMSO-d_6_) δ 1.26 (t, *J* = 6.8 Hz, 2H, OCH_2_CH_3_), 3.29 (s, 3H, SO_2_CH_3_), 4.23 (q, *J* = 6.8 Hz, 3H, OCH_2_CH_3_), 7.54 (d, *J* = 8.4 Hz, 2H, phenyl H-2, H-6), 7.86 (d, *J* = 8.4 Hz, 2H, phenyl H-3, H-5), 7.89 (m, 1H, phenyl H-4), 8.09 (d, *J* = 8.4 Hz, 2H, aminosulfonylphenyl H-3, H-5), 8.29 (d, *J* = 8.4 Hz, 2H, aminosulfonlphenyl H-2, H-6), 9.34 (s, 1H, pyrazole H-5); ^13^C NMR (DMSO-d_6_) δ 14.58, 43.99, 60.75, 114.60, 119.96, 128.48, 129.29, 130.83, 131.42, 134.21, 134.99, 139.63, 142.48, 152.78, 162.45; MS (m/z, relative abundance %): 370.10 (M +) (100%). Anal. Calcd. For C_19_H_18_N_2_O_4_S: C, 61.61; H, 4.90; N, 7.56; Found; C, 61.98; H, 5.12; N, 7.88.

#### Ethyl 1-(4-(methylsulfonyl)phenyl)-3-(p-tolyl)-1*H*-pyrazole-5-carboxylate (19b)

Yield 71%; white solid; m.p. 267–269 °C; IR (KBr disk) 1693 (C = O), 1355, 1149 (SO_2_); ^1^H NMR (DMSO-d_6_) δ 1.26 (t, *J* = 6.8 Hz, 2H, OCH_2_CH_3_), 2.37 (s, 3H, CH_3_), 3.28 (s, 3H, SO_2_CH_3_), 4.22 (q, *J* = 6.8 Hz, 3H, OCH_2_CH_3_), 7.26 (d, *J* = 8.0 Hz, 2H, 4-methylphenyl H-3, H-5), 7.70 (d, *J* = 8.4 Hz, 2H, 4-methylphenyl H-2, H-6), 8.07 (d, *J* = 8.4 Hz, 2H, aminosulfonylphenyl H-3, H-5), 8.26 (d, *J* = 8.4 Hz, 2H, aminosulfonlphenyl H-2, H-6), 9.28 (s, 1H, pyrazole H-5); ^13^C NMR (DMSO-d_6_) δ 14.60, 21.40, 44.01, 60.62, 119.83, 128.94, 129.28, 129.45, 129.49, 134.72, 138.85, 139.40, 142.57, 142.66, 153.95, 162.59; MS (m/z, relative abundance %): 384.11 (M +) (100%). Anal. Calcd. For C_20_H_20_N_2_O_4_S: C, 62.48; H, 5.24; N, 7.29; Found; C, 62.64; H, 5.38; N, 7.58.

#### Ethyl 3-(4-chlorophenyl)-1-(4-(methylsulfonyl)phenyl)-1*H*-pyrazole-5-carboxylate (19c)

Yield 76%; white solid; m.p. 285–287 °C; IR (KBr disk) 1701 (C = O), 1355, 1149 (SO_2_); ^1^H NMR (DMSO-d_6_) δ 1.26 (t, *J* = 6.8 Hz, 2H, OCH_2_CH_3_), 3.29 (s, 3H, SO_2_CH_3_), 4.23 (q, *J* = 6.8 Hz, 3H, OCH_2_CH_3_), 7.53 (d, *J* = 8.8 Hz, 2H, 4-chlorophenyl H-2, H-6), 7.85 (d, *J* = 8.8 Hz, 2H, 4-chlorophenyl H-3, H-5), 8.08 (d, *J* = 8.8 Hz, 2H, aminosulfonylphenyl H-3, H-5), 8.28 (d, *J* = 8.8 Hz, 2H, aminosulfonlphenyl H-2, H-6), 9.33 (s, 1H, pyrazole H-5); ^13^C NMR (DMSO-d_6_) δ 14.57, 43.99, 60.76, 114.60, 119.96, 128.47, 129.29, 130.82, 131.42, 134.20, 134.97, 139.61, 142.47, 152.77, 162.45; MS (m/z, relative abundance %): 406.06 (M +) (100%). Anal. Calcd. For C_19_H_17_ClN_2_O_4_S: C, 56.36; H, 4.23; N, 6.92; Found; C, 56.44; H, 4.48; N, 7.15.

#### Ethyl 3-(4-bromophenyl)-1-(4-(methylsulfonyl)phenyl)-1*H*-pyrazole-5-carboxylate (19d)

Yield 66%; white solid; m.p. 293–295 °C; IR (KBr disk) 1693 (C = O), 1355, 1141 (SO_2_); ^1^H NMR (DMSO-d_6_) δ 1.26 (t, *J* = 7.2 Hz, 2H, OCH_2_CH_3_), 3.28 (s, 3H, SO_2_CH_3_), 4.23 (q, *J* = 7.2 Hz, 3H, OCH_2_CH_3_), 7.66 (d, *J* = 8.4 Hz, 2H, 4-bromophenyl H-2, H-6), 7.78 (d, *J* = 8.4 Hz, 2H, 4-bromophenyl H-3, H-5), 8.08 (d, *J* = 8.8 Hz, 2H, aminosulfonylphenyl H-3, H-5), 8.27 (d, *J* = 8.8 Hz, 2H, aminosulfonlphenyl H-2, H-6), 9.32 (s, 1H, pyrazole H-5); ^13^C NMR (DMSO-d_6_) δ 14.56, 44.00, 60.79, 114.58, 119.96, 122.91, 129.30, 131.15, 131.39, 131.67, 134.94, 139.57, 142.46, 152.84. 162.45; MS (m/z, relative abundance %): 448.01 (M +) (100%). Anal. Calcd. For C_19_H_17_BrN_2_O_4_S: C, 50.79; H, 3.81; N, 6.23; Found; C, 50.54; H, 3.52; N, 6.14.

### High performance liquid chromatography (HPLC) purity analysis for the final most potent compounds 15b, 15e, 15f, 19b and 19c

The percent purity of the final compounds **15b, 15e, 15f, 19b** and** 19c** was determined through high performance liquid chromatography (HPLC) analysis using (Agilent HPLC Model No. 1100, Serial No. DEO3010942 utilizing GL Sciences Inertsil ph-3–5 µm, 4.6 × 250 mm column) (Cat. No. 5020–01921, Serial No. TH5-5726, Japan) at Faculty of Graduate Studies for Advanced Sciences, Beni-Suef University, using buffer: 2.7 g/L of monobasic potassium phosphate adjusted with phosphoric acid to a pH of 3.0 and mobile phase: methanol, acetonitrile and buffer (3:1:6) at 1.5 mL/min flowrate, sample solution is 0.5 mg/mL of each compound **16d** and** 19b** in diluent (methanol/water 3:1). HPLC chromatograms for compounds **15b, 15e, 15f, 19b** and** 19c** were provided in supplementary section with all detailed reports which showed that retention time of solvent mobile phase was 1.738 min. and that for compounds **15b, 15e, 15f, 19b** and** 19c** were 2.164, 2.419, 2.418, 2.417 and 2.413 min, respectively. Solvent mobile phase chromatogram was provided to establish a base line for comparison. It was found that the purity of these compounds was almost 97.9–98.9%.

## Biological evaluation

### In vitro anti-inflammatory activity against COX-1 and COX-2 enzymes

Determination of the in vitro COX-1/COX-2 inhibition was carried out using COX-1 Inhibitor Screening Kit-K548 (Biovision, S. Milpitas Blvd., Milpitas, CA 95035 USA) and COX-2 Inhibitor Screening Kit-K547 (Biovision, S. Milpitas Blvd., Milpitas, CA 95035 USA). The IC_50_ value of the tested compounds was determined. Moreover, the COX-2 selectivity index (S.I values), which are calculated using the formula IC_50_ (COX-1)/IC_50_ (COX-2), were determined and compared to that of the used reference celecoxib (as a selective COX-2 inhibitor).

**COX-1 inhibitor Screening Protocol:** Dissolve the tested compounds in DMSO. Use COX assay buffer to dilute the relevant test concentration by 10X before using. Place 10 µl of diluted test inhibitor or assay buffer in the specified wells instead of the sample screen (S) or enzyme control (EC; no inhibitor). To one of the wells, add 2 µl of SC560 and 8 µl of COX Assay Buffer as an inhibitor control (IC). Before using, combine 2 µl of COX Cofactor with 398 µl of COX Assay Buffer to dilute COX cofactor 200 times. Blend thoroughly. Just before using, mix 5 µl of the provided arachidonic acid with 5 µl of NaOH to make the arachidonic acid solution. Combine quickly using a vortex. To dilute the arachidonic acid/NaOH solution 10 times, add 90 µl of water. There should be 80 µl of reaction mix in each well. Using a multi-channel pipette, add 10 µl of diluted arachidonic acid/NaOH solution to each well to initiate all the reactions at once. Fluorescence (Ex/Em = 535/587 nm) measured kinetically at 25 °C for 5–10 min.

**COX-2 inhibitor Screening Protocol:** Dissolve the tested compounds in DMSO. Use COX Assay Buffer to dilute the relevant test concentration by 10X before using. Place 10 µl of diluted test inhibitor or Assay Buffer in the specified wells instead of the sample screen (S) or enzyme control (EC; no inhibitor). To one of the wells, add 2 µl of celecoxib and 8 µl of COX Assay Buffer as an inhibitor control (IC). Before using, combine 2 µl of COX Cofactor with 398 µl of COX Assay Buffer to dilute COX Cofactor 200 times. Blend thoroughly. Just before using, mix 5 µl of the provided arachidonic acid with 5 µl of NaOH to make the arachidonic acid solution. Combine quickly using a vortex. To dilute the arachidonic acid/NaOH solution 10 times, add 90 µl of water. There should be 80 µl of reaction mix in each well. Using a multi-channel pipette, add 10 µl of diluted arachidonic acid/NaOH solution into each well to initiate the reactions concurrently. Fluorescence (Ex/Em = 535/587 nm) measured kinetically at 25 °C for 5–10 min.

### In vitro anti-inflammatory activity against 5-LOX/15-LOX enzymes

The in vitro 5-LOX/15-LOX inhibition was assessed using the 5-lipoxygenase Inhibitor Screening Kit (Catalog # K980-100) from Biovision, S. Milpitas Blvd., Milpitas, CA 95035 USA and the Lipoxygenase Inhibitor Screening assay Kit (Item # 760,700) from Cayman Chemical, 1180, East Ellsworth Rd., Ann Arbor, MI 48108, USA [49, 50]. The test compounds potency was determined by calculating the concentration that inhibits an enzyme by 50% (IC_50_).

**5-LOX inhibitor Screening Protocol:** dissolve the test compounds in DMSO. The solution was prepared at a concentration so that the final 100 µl reaction volume per well contains no more than 2 µl of the test chemical solution added to it. Each well was filled in the 96-well white plate with 2 µl of the test substance. In test wells, 2 µl of the solvent used was added to generate the test compound solution at its final concentration for the “Solvent Control” and 2 µl of the supplied LOX inhibitor, zileuton, for the “Inhibitor Control.” 38 µl of LOX Assay Buffer was added to each well to increase the volume to 40 µl. To the “Enzyme Control” well, was filled with 40 µl of LOX Assay Buffer. In order to run the required number of assays, enough reagents were mixed. 40 µl of the following mix was prepared for each well: Buffer for Reaction MIx LOX Assay 34 µl LOX Probe 2 µl 5. LOX Enzyme 4 µl After thoroughly mixing, the Reaction Mix was transferred to the wells holding the Test Compounds, Enzyme Control, Inhibitor Control, and “Solvent Control.” substrate was added after 10 min of RT incubation of the plate. The wells shouldn’t contain any bubbles. To create a 500 X solution, the supplied LOX substrate was diluted (12,500 X) in LOX Assay Buffer using a 1:25 dilution factor. To obtain the 5X solution, the 500 X solution with LOX Assay Buffer was diluted at a ratio of 1:100, depending on the number of reactions to be conducted. For each reaction, 20 µl of 5X solution will be required. How much substrate is needed based on the number of reactions. The final substrate functioning solution needs to be consumed that same day and stored on ice. The remaining stock solution should be immediately stored at -20ºC. 20 µl of 5X LOX Substrate should be added with a multichannel pipette to every well. The second minute after the substrate is added; begin recording fluorescence at Ex/Em 500/536 nm at 30-s intervals for 30 to 60 min. To get the RFU for each sample, the RFU was subtracted at time t1 from the RFU at time t2, making sure that both t2 and t1 fall within the assay's linear range. The slope for each sample (including the “enzyme control”)was multiplied by the time Δt (t2 – t1) to find the ΔRFU. In the equations below, the values was substituted for “Solvent Control” for “Enzyme Control” if the two slopes disagree.

$$\% Inhibition=[slope of (enzyme control)-slope of (test compound)]/slope of (enzyme control)\times 100$$

$$\% Relative activity=[slope (test compound)]/slope (enzyme control)\times 100$$

**15-LOX inhibitor screening protocol:** Blank wells: 100 µl of assay buffer was added to at least two wells, Positive control wells: 90 µl of 15-LOX standard was added to 10 µl of assay buffer to at least two wells, 100% initial activity wells: 90 µl of Lipoxygenase enzyme was added to 10 µl of solvent to at least two wells, Inhibitor wells: 90 µl of Lipoxygenase enzyme was added to 10 µl of inhibitor wells to. It was incubated for five minutes at room temperature. The reaction was initiated by adding 10 µl of substrate to all the wells. 96well-plates were placed on a shaker for at least 10 min. 100 µl of chromogen was added to each well to stop the enzyme catalysis. The reaction was covered with plate cover. 96well-plates were placed on a shaker for at least 5 min. The cover was removed and absorbance was read using plate reader at absorbance 490–500 nm.

### TNF-α and PEG2 productions in LPS-activated RAW 264.7 macrophages

The inhibition of LPS-induced inflammation via attenuating **TNF-α** and **PGE2** cytokines production was investigated for compounds **15c, 15d, 15 h** and** 19d** as detailed in previous procedure [9, 57]. Briefly, LPS (1 µg/mL) was added for an additional 20 h. after RAW 264.7 macrophages had been incubated with compounds **15c, 15d, 15 h** and** 19d** in concentrations of (12.5, 25, and 50 µM) for two h. Following the manufacturer's instructions, TNF-α (ab181421 Human TNF alpha Simple Step ELISA® Kit, Abcam Inc. 152 Grove Street Waltham, MA 02453 USA) and PEG2 (Catalog Number KGE004B, Inc., MN, USA) IC_50_ were measured in the cell culture supernatants using these commercially available ELISA kits [9, 29, 57, 58]**.**

### Inducible nitric oxide synthase (iNOS) inhibition

NO is produced from L-arginine by iNOS and is utilized in a variety of cell signaling processes. An inducible member of the NOS family, iNOS is increased during pro-inflammatory cytokine activity as a host-defense mechanism. A commercially available quantitative ELISA kit (ab253219 Mouse iNOS Simple Step ELISA® Kit) will be used to evaluate the iNOS enzyme activity of the substances in the cell lysate in accordance with the manufacturer’s instructions [59].

### Cell culture and cytotoxicity assay

Using Dulbecco’s Modified Eagle Medium (DMEM, Biowest L0060) supplemented with 10% foetal bovine serum (FBS) (Biowest, S1810) and 1% anti-biotic and anti-mycotic (Biowest, L0010-100), the RAW264.7 macrophage cell line (ATCC ® TIB-71TM) was cultivated. The cells were then incubated at 37 °C with 5% CO_2_ until they were confluent, which should be between 70 and 80%. After two to three days, the growth media was regularly changed. Trypsin–EDTA was then used to wash and collect the cells (Biowest, L0931-500).

The MTS (3-(4,5-dimethylthiazol-2-yl)-5-(3-carboxymethoxy-phenyl)-2-(4-sulfophenyl)-2H-tetrazolium) test (Promega, ab197010) was used to determine cell viability. In short, a 96-well plate was seeded with 5 × 103 cells per well, and the cells were then incubated for 24 h at 37 °C and 5% CO_2_. The cells were then re-incubated for 24 h at 37 °C and 5% CO_2_ after the medium was discarded and replaced with 180 µl of fresh growth media and 20 µl of sample (1000 ug, 250 ug, 63 ug, 16 ug, and 4 ug µg/mL). 20 µl of MTS was then added to each well, and the mixture was incubated for three hours at 37 °C with 5% CO_2_. A spectrophotometer (Multiskan GO Thermo Scientific 51,119,300, Thermo Fisher Scientific, USA) was used to detect the absorbance at 490 nm. Control cells were those that received no treatment [60].

#### In vivo anti-inflammatory activity

Using an in vivo carrageenan-induced rat foot paw edema model, the anti-inflammatory properties of the most effective compounds **15c, 15d, 15 h** and** 19d** were assessed. As previously described [35], paw thickness was evaluated at 1, 3, and 5 h following carrageenan injection at a dose of 50 mg/kg. Furthermore, using the procedures outlined in [35], the dose at which 50% edema inhibition (ED_50_) was achieved for the strongest anti-inflammatory compounds **15c, 15d, 15 h** and** 19d** was determined in relation to celecoxib.

### Ulcerogenic liability

The ulcerogenic effect of **15c, 15d, 15 h, 19d**, celecoxib and lonazolac was determined using the previously reported procedures [49]. Rats were divided into 7 groups and fasted for 18 h before drug administration. The control group received the vehicle (2.5% Tween 80). Other groups were received test compounds, celecoxib or lonazolac as reference drugs at a dose of 50 mg/kg. After 2 h, animals were fed. Rats were given the required dose orally for three successive days. After 2 h of the last dose, rats were sacrificed; the stomach of each rat was removed then opened along the greater curvature and rinsed with saline. In order to examine the stomach, it was stretched by pins on a corkboard. The gastric mucosa was carefully inspected for the occurrence of ulcers with the aid of an illuminated magnifying lens (l0x), then ulcer index was calculated according to the method described by Cho and Ogle [51]. Lesions were counted and measured along the greater diameter using transparent ruler. Every five hemorrhagic spots were considered equivalent to 1 mm of ulcer. The ulcer index (mm) was calculated from the sum of the total length of ulcers and hemorrhagic spots in each stomach.

## Molecular modeling study

Docking was performed to secure a confirmation and binding energy ranking prediction between target enzymes and new designed compounds using Molecular Operating Environment (MOE) version 2015.10 modeling software. All docking studies were carried out using enzyme downloaded from Protein Data Bank (PDB). The crystal structure of the reference drug celecoxib and zileuton bound at the COX-2 and 5-LOX active site obtained from protein data bank at Research Collaboration for Structural Bioinformatics (RSCB) protein data bank COX-2 enzyme (PDB ID:3LN1) and 5-LOX enzyme (PDB ID: 3V99) The co-crystallized ligand (celecoxib) in COX-2 and zileuton in 5-LOX receptors were docked firstly to study its energy score, root mean standard deviation (RMSD) and interaction of different amino acids. The best crystal-like poses of them were analyzed and the least energetic conformer was detected. London DG force and force field energy were used for the refinement of results. Docking of the tested compound was executed after their 3D protonation using the 3D structure built by MOE, running conformational analysis then selecting the least energetic conformer. The same protocol used for ligands and designed compounds after minimizing energy was performed. Poses displaying the best superimposition mode on the ligand and binding energy score for each compound were analyzed to identify potential interactions with amino acids in the active site of each enzyme.

Results that obtained from docking amino acid interactions, hydrogen bond lengths and energy scores were summarized in Tables 6 and 7.

## Estimation of in silico ADME properties

The ADME study was performed by using the SwissADME web tool (http://www.swissadme.ch) after drawing the compounds by Chem Sketch (v.12) and converting them to SMILE to examine the ability of the molecule to be used as a drug. It predicts the physiochemical properties, absorption, distribution, metabolism, elimination, and pharmacokinetic properties of molecules, so it is considered the key endeavor to further clinical trials. The determined parameters which were established to the most selective compounds **15c, 15d, 15 h** and** 19d** in addition to celecoxib were listed in (Table 8).

## Conclusion

As new non-acidic lonazolac analogues, two new series of pyrazole ester derivatives, **15a-h** and **19a-d**, were designed, synthesized and evaluated for their anti-inflammatory activity. The most potent derivatives **15c, 15d, 15 h**, and **19d**, showed COX-2 selectivity index in the range of (28.56–98.71) when compared to celecoxib (S.I. = 13.65). Additionally, the four most powerful derivatives shown exceptional 5-LOX and 15-LOX inhibitory actions (IC_50_ = 0.24–0.81 µM, 0.20–2.2 µM, respectively), in comparison to zileuton. Upon using lipopolysaccharide-activated RAW 264.7 macrophages, derivatives **15c, 15d, 15 h**, and **19d** showed similar inhibitory activities against TNF-α and PGE2 (IC_50_ = 0.77–1.20 μM and 0.28–0.52 μM, respectively) compared to celecoxib (IC_50_ = 0.87 μM and 0.38 μM, respectively) as a reference compound. Compounds **15c, 15d, 15 h** and** 19d** remarkably shown greater inhibition of inducible nitric oxide synthase (iNOS) with lower IC_50_ (0.41–0.61 µM), when compared to the reference drug celecoxib (0.48 µM). Additionally, the results indicated that compounds **15c, 15d, 15 h,** and** 19d** were less cytotoxic and safer, with greater IC_50_ values (178.95–301.40 µM) than the reference drug, celecoxib (148.90 µM). Concerning in vivo anti-inflammatory activity, these four compounds (ED_50_ = 8.22–31.22 mg/kg, respectively) were more effective than celecoxib (ED_50_ = 40.39 mg/kg). Finally, in comparison to lonazolac (ulcer index = 20.30) and celecoxib (ulcer index = 3.02), all of the screened derivatives **15c, 15d, 15 h**, and **19d** were less ulcerogenic (ulcer indexes = 1.22–2.93).

Finally, it is clear that; substitution of the acidic moiety of lonazolac with the ethyl ester one in the target compounds **15a-h** and **19a-d** resulted in increasing the safety profiles of these analogues. Also, the incorporation of SO_2_CH_3_-COX-2 selective inhibition pharmacophore in the para position of the pyrazole-*N*-1-phenyl moiety achieved the COX-2 selectivity of these analogues over that of lonazolac.

## Data Availability

No datasets were generated or analysed during the current study.

## References

[1] Chen L, Deng H, Cui H, Fang J, Zuo Z, Deng J, Li Y, Wang X, Zhao L (2018) Inflammatory responses and inflammation-associated diseases in organs. Oncotarget 9(6):7204. 10.18632/oncotarget.23208 29467962 10.18632/oncotarget.23208 PMC5805548

[2] Ohshima H, Tatemichi M, Sawa T (2003) Chemical basis of inflammation-induced carcinogenesis. Arch Biochem Biophys 417(1):3–11. 10.1016/s0003-9861(03)00283-2 12921773 10.1016/s0003-9861(03)00283-2

[3] Qian B-Z (2017) Inflammation fires up cancer metastasis. Semin Cancer Biol 47:170–176. 10.1016/j.semcancer.2017.08.006 28838845 10.1016/j.semcancer.2017.08.006

[4] Ma L, Pei H, Lei L, He L, Chen J, Liang X, Peng A, Ye H, Xiang M, Chen L (2015) Structural exploration, synthesis and pharmacological evaluation of novel 5-benzylidenethiazolidine-2,4-dione derivatives as iNOS inhibitors against inflammatory diseases. Eur J Med Chem 92:178–190. 10.1016/j.ejmech.2014.12.036 25555141 10.1016/j.ejmech.2014.12.036

[5] Abdulkhaleq LA, Assi MA, Abdullah R, Zamri-Saad M, Taufiq-Yap YH, Hezmee MNM (2018) The crucial roles of inflammatory mediators in inflammation: a review. Vet World 11(5):627–635. 10.14202/vetworld.2018.627-635 29915501 10.14202/vetworld.2018.627-635 PMC5993766

[6] Fadaly WAA, Elshaier YAMM, Nemr MTM, Abdellatif KRA (2023) Design, synthesis, modeling studies and biological evaluation of pyrazole derivatives linked to oxime and nitrate moieties as nitric oxide donor selective COX-2 and aromatase inhibitors with dual anti-inflammatory and anti-neoplastic activities. Bioorg Chem. 10.1016/j.bioorg.2023.106428 10.1016/j.bioorg.2023.106428 36893546

[7] Chakraborty K, Krishnan S, Joy M (2021) Euryfuranyl compounds from edible species of cuttlefish as potential anti-inflammatory leads attenuating NF-κB signaling cascade in lipopolysaccharide-activated macrophages. Bioorg Chem. 10.1016/j.bioorg.2021.105052 34146918 10.1016/j.bioorg.2021.105052

[8] Liu Z, Zhang W, Zhang M, Zhu H, Moriasi C, M.-Hui Zou, (2015) Liver kinase B1 suppresses lipopolysaccharide-induced nuclear factor κB (NF-κB) activation in macrophages. J Bio Chem 23:2312–2320. 10.1074/jbc.M114.616441 10.1074/jbc.M114.616441 PMC4303683 25451940

[9] El-Din MMG, El-Gamal MI, Abdel-Maksoud MS, Lee H, Choi J, Kim T, Shin J, Lee H, Kim H, Lee K, Baek D (2020) Inhibitory effects of triarylpyrazole derivatives on LPS-induced nitric oxide and PGE2 productions in murine RAW 264.7 macrophages. Bioorg Med Chem Lett 30:126884. 10.1016/j.bmcl.2019.126884 31879211 10.1016/j.bmcl.2019.126884

[10] Fadaly WAA, Nemr MTM, Kahk NM (2024) Discovery of novel pyrazole based urea/thiourea derivatives as multiple targeting VEGFR-2, EGFR^WT^, EGFR^T790M^ tyrosine kinases and COX-2 inhibitors, with anti-cancer and anti-inflammatory activities. Bioorg Chem 147:107403. 10.1016/j.bioorg.2024.107403 38691909 10.1016/j.bioorg.2024.107403

[11] Hegazy ME, Taher ES, Ghiaty AH, Bayoumi AH (2024) Tailored quinoline hybrids as promising COX-2/15-LOX dual inhibitors endowed with diverse safety profile: design, synthesis, SAR, and histopathological study. Bioorg Chem 145:107244. 10.1016/j.bioorg.2024.107244 38428284 10.1016/j.bioorg.2024.107244

[12] Bhuktar H, Shukla S, Kakularam KR, Srikanth Battu M, Srikanth S, Srivastava R. Medishe, Ram P, Jagadish PC, Rasool M, Chakraborty S, Khan N, Reddanna P, Oruganti S, Pal M (2023) Design, synthesis and evaluation of 2-aryl quinoline derivatives against 12R-lipoxygenase (12R-LOX): discovery of first inhibitor of 12R-LOX. Bioorg Chem 138:106606. 10.1016/j.bioorg.2023.106606 37210826 10.1016/j.bioorg.2023.106606

[13] Z. Nawaz, N. Riaz, M. Saleem, A. Iqbal, S. AbidaEjaz, B. Bashir, S. Muzaffar, M. Ashraf, A.-ur-Rehman, M. S. Bilal, B. K. Prabhala, S. Sajid., Molecular hybrids of substituted phenylcarbamoylpiperidine and 1,2,4-triazole methylacetamide as potent 15-LOX inhibitors: Design, synthesis, DFT calculations and molecular docking studies. Bioorg. Chem. 143 (2024) 106984. 10.1016/j.bioorg.2023.106984 10.1016/j.bioorg.2023.106984 38056389

[14] Kilty I, Logan A, Vickers PJ (1999) Differential characteristics of human 15-lipoxygenase isozymes and a novel splice variant of 15S-lipoxygenase. Eur J Biochem 266:83–93. 10.1046/j.1432-1327.1999.00818.x 10542053 10.1046/j.1432-1327.1999.00818.x

[15] Brash AR, Boeglin WE, Chang MS (1997) Discovery of a second 15S-lipoxygenase in humans. Proc Natl Acad Sci USA 94:6148–6152. 10.1073/pnas.94.12.6148 9177185 10.1073/pnas.94.12.6148 PMC21017

[16] Mitoma H, Horiuchi T, Tsukamoto H, Ueda N (2018) Molecular mechanism of action of anti-TNF- α agents-comparison among therapeutic TNF-α antagonists. Cytokine 101:56–63. 10.1016/j.cyto.2016.08.014 27567553 10.1016/j.cyto.2016.08.014

[17] Mohamed MFA, Marzouk AA, Nafady A, El-Gamal DA, Allam RM, Abuo-Rahma G-E-D-A, El Subbagh HI, Moustafa AH (2020) Design, synthesis and molecular modeling of novel aryl carboximidamides and 3-aryl-1,2,4-oxadiazoles derived from indomethacin as potent anti-inflammatory iNOS/PGE2 inhibitors Bioorg. Chem 105:104439. 10.1016/j.bioorg.2020.104439 10.1016/j.bioorg.2020.104439 33161252

[18] Stuehr DJ, Santolini J, Wang ZQ, Wei CC, Adak S (2004) Update on mechanism and catalytic regulation in the NO synthases. J Biol Chem 279(35):36167–36170. 10.1074/jbc.R400017200 15133020 10.1074/jbc.R400017200

[19] Abdellatif KRA, Fadaly WAA, Elshaier Y, Ali WAM, Kamel GM (2018) Non-acidic 1,3,4-trisubstituted-pyrazole derivatives as lonazolac analogs with promising COX-2 selectivity, anti-inflammatory activity and gastric safety profile. Bioorg Chem 77:568–578. 10.1016/j.bioorg.2018.02.018 29475165 10.1016/j.bioorg.2018.02.018

[20] Abdellatif KR, Fadaly WAA, Ali WA, Kamel GM (2016) Synthesis, cyclooxygenase inhibition, anti-inflammatory evaluation and ulcerogenic liability of new 1,5diarylpyrazole derivatives. J Enzyme Inhib Med Chem 31:54–60. 10.1080/14756366.2016.1201815 27541738 10.1080/14756366.2016.1201815

[21] Abdellatif KR, Fadaly WAA, Azouz AA (2016) Synthesis, Cyclooxygenase inhibition, anti-inflammatory evaluation, and ulcerogenic liability of new 1,3,5-triarylpyrazoline derivatives possessing a methanesulfonyl pharmacophore. Arch Pharm 349(10):801–807. 10.1002/ardp.201600145 10.1002/ardp.201600145 27601359

[22] Fadaly WAA, Elshaier YAMM, Hassanein EHM, Abdellatif KRA (2020) New 1,2,4-triazole/pyrazole hybrids linked to oxime moiety as nitric oxide donor celecoxib analogs: Synthesis, cyclooxygenase inhibition anti-inflammatory, ulcerogenicity, anti-proliferative activities, apoptosis, molecular modeling and nitric oxide release studies. Bioorg Chem 98:103752. 10.1016/j.bioorg.2020.103752 32197148 10.1016/j.bioorg.2020.103752

[23] Fadaly WAA, Elshaier YAMM, Nemr MTM, Abdellatif KRA (2023) Design, synthesis, modeling studies and biological evaluation of pyrazole derivatives linked to oxime and nitrate moieties as nitric oxide donor selective COX-2 and aromatase inhibitors with dual anti-inflammatory and anti-neoplastic activities. Bioorg Chem 134:106428. 10.1016/j.bioorg.2023.106428 36893546 10.1016/j.bioorg.2023.106428

[24] Abdellatif KRA, Abdelall EKA, Fadaly WAA, Kamel GM (2016) Synthesis, cyclooxygenase inhibition, anti-inflammatory evaluation and ulcerogenic liability of new 1,3,5-triarylpyrazoline and 1,5-diarylpyrazole derivatives as selective COX-2 inhibitors. Bioorg Med Chem Lett 26:406–412. 10.1016/j.bmcl.2015.11.105 26691756 10.1016/j.bmcl.2015.11.105

[25] Abdellatif KRA, Abdelall EKA, Labib MB, Fadaly WAA, Zidan TH (2020) Synthesis of novel halogenated triarylpyrazoles as selective COX-2 inhibitors: anti-inflammatory activity, histopatholgical profile and in-silico studies. Bioorg Chem 105:104418. 10.1016/j.bioorg.2020.104418 33166844 10.1016/j.bioorg.2020.104418

[26] Abdelgawad MA, Labib MB, Abdel-Latif M (2017) Pyrazole-hydrazone derivatives as anti-inflammatory agents: design, synthesis, biological evaluation, COX-1,2/ 5-LOX inhibition and docking study. Bioorg Chem 74:212–220. 10.1016/j.bioorg.2017.08.014 28865292 10.1016/j.bioorg.2017.08.014

[27] Abdelgawad MA, Labib MB, Ali WAM, Kamel G, Azouz AA, E. EL-Nahass, (2018) Design, synthesis, analgesic, anti-inflammatory activity of novel pyrazolones possessing aminosulfonyl pharmacophore as inhibitors of COX-2/5-LOX enzymes: histopathological and docking studies. Bioorg Chem 78:103114. 10.1016/j.bioorg.2018.03.011 10.1016/j.bioorg.2018.03.011 29550530

[28] Ahmed AHH, Mohamed MFA, Allam RM, Nafady A, Mohamed SK, Gouda AE, Beshr EAM (2022) Design, synthesis, and molecular docking of novel pyrazole-chalcone analogs of lonazolac as 5-LOX, iNOS and tubulin polymerization inhibitors with potential anticancer and anti-inflammatory activities. Bioorg Chem 129:106171. 10.1016/j.bioorg.2022.106171 36166898 10.1016/j.bioorg.2022.106171

[29] Hwang JH, Ma JN, Park JH, Jung HW, Park YK (2019) Anti-inflammatory and antioxidant effects of MOK, a polyherbal extract, on lipopolysaccharide stimulated RAW 264.7 macrophages. Int J Mol Med 43(1):26–36. 10.3892/ijmm.2018.3937 30365058 10.3892/ijmm.2018.3937 PMC6257867

[30] Abdellatif KR, Fadaly WAA, Mostafa YA, Zaher DM, Omar HA (2019) Thiohydantoin derivatives incorporating a pyrazole core: design, synthesis and biological evaluation as dual inhibitors of topoisomerase-I and cycloxygenase-2 with anti-cancer and anti-inflammatory activities. Bioorg Chem 91:103132. 10.1016/j.bioorg.2019.103132 31374529 10.1016/j.bioorg.2019.103132

[31] Fadaly WAA, Zidan TH, Kahk NM, Mohamed FEA, Abdelhakeem MM, Khalil RG, Nemr MTM (2023) New pyrazolyl-thiazolidinone/thiazole derivatives as celecoxib/dasatinib analogues with selective COX-2, HER-2 and EGFR inhibitory effects: design, synthesis, anti-inflammatory/anti-proliferative activities, apoptosis, molecular modeling and ADME studies. J Enzyme Inhib Med Chem 38(1):2281262. 10.1080/14756366.2023.2281262 38010912 10.1080/14756366.2023.2281262 PMC11003491

[32] Fadaly WAA, Nemr MTM, Zidan TH, Mohamed FEA, Abdelhakeem MM, Abu Jayab NN, Omar HA, Abdellatif KRA (2023) New 1,2,3-triazole/1,2,4-triazole hybrids linked to oxime moiety as nitric oxide donor selective COX-2, aromatase, B-RAFV600E and EGFR inhibitors celecoxib analogs: design, synthesis, anti-inflammatory/anti-proliferative activities, apoptosis and molecular modeling study. J Enzym Inhib Med Chem 38(1):2290461. 10.1080/14756366.2023.2290461 10.1080/14756366.2023.2290461 PMC11003496 38061801

[33] Fadaly WAA, Nemr MTM, Abd El-Hameed AM, Giovannuzzi S, Alkabbani MA, Hefina MM, Nocentini A, Supuran CT, Eldehna WM, Mohamed MFA, Zidan TH (2025) Novel 1,2,3-triazole/imino-thiazolidinone benzenesulfonamide derivatives linked to diaryl pyrazole tail as potential carbonic anhydrase II/VII Inhibitors with anti-epileptic activity. Eur J Med Chem 291:117619. 10.1016/j.ejmech.2025.117619 40249969 10.1016/j.ejmech.2025.117619

[34] Fadaly WAA, Elshaier Yaseen A. M. M, Ali Fares E. M, El-Bahrawy Ali H, Abdellatif Khaled R. A, Nemr Mohamed T. M (2024) Vicinal diaryl pyrazole with tetrazole/urea scaffolds as selective angiotensin converting enzyme-1/cyclooxygenase-2 inhibitors: design, synthesis, anti-hypertensive, anti-fibrotic, and anti-inflammatory. Drug Dev Res 85(4):e22217. 10.1002/ddr.22217 38845214 10.1002/ddr.22217

[35] Fadaly WAA, Elshewy A, Nemr MTM, Abdou K, Sayed AM, Kahk NM (2024) Discovery of novel thiazole derivatives containing pyrazole scaffold as PPAR-γ Agonists, α-Glucosidase, α-Amylase and COX-2 inhibitors; design, synthesis and in silico study. Bioorg Chem 152:107760. 10.1016/j.bioorg.2024.107760 39197383 10.1016/j.bioorg.2024.107760

[36] Nemr MTM, AboulMagd AM (2020) New fused pyrimidine derivatives with anticancer activity: synthesis, topoisomerase II inhibition, apoptotic inducing activity and molecular modeling study. Bioorg Chem. 10.1016/j.bioorg.2020.104134 32750610 10.1016/j.bioorg.2020.104134

[37] Nemr MTM, Sonousi A, Marzouk AA (2020) Design, synthesis and antiproliferative evaluation of new tricyclic fused thiazolopyrimidines targeting topoisomerase II: molecular docking and apoptosis inducing activity. Bioorg Chem. 10.1016/j.bioorg.2020.104446 33171405 10.1016/j.bioorg.2020.104446

[38] Abdalla M, Elmasry AE, Nemr MTM, Alanazi MM, Attallah NGM, Elshaier YAAM (2025) Insight study for repurposing of certain anti-inflammatory drugs based on aspirin and salicylic acid scaffolds for the treatment of cancer as CDKs inhibitors: cheminformatics and anticancer studies. ChemistrySelect 10(15):e202500175. 10.1002/slct.202500175

[39] Nemr MTM, Teleb M, AboulMagd AM, El-Naggar ME, Gouda N, Abdel-Ghany AA, Elshaier YAMM (2023) Design, synthesis and chemoinformatic studies of new thiazolopyrimidine derivatives as potent anticancer agents via phosphodiesterase-5 inhibition and apoptotic inducing activity. J Mol Struct 1272:134216. 10.1016/j.molstruc.2022.134216

[40] Nemr MTM, Elshewy A, Labib M, El Kerdawy AM, Halim PA (2024) Design, synthesis, antineoplastic activity of new pyrazolo [3, 4-d] pyrimidine derivatives as dual CDK2/GSK3β kinase inhibitors; molecular docking study, and ADME prediction. Bioorg Chem 150:107566. 10.1016/j.bioorg.2024.107566 38896936 10.1016/j.bioorg.2024.107566

[41] AbdEl-Mawgoud HK, AboulMagd AM, Nemr MTM, Hemdan MM, Hassaballah AI, Farag PS (2024) Design, synthesis and cytotoxic evaluation of new thieno[2,3-d]pyrimidine analogues as VEGFR-2/AKT dual inhibitors, apoptosis and autophagy inducers. Bioorg Chem 150:107622. 10.1016/j.bioorg.2024.107622 38996545 10.1016/j.bioorg.2024.107622

[42] Moustafa AH, AboulMagd AM, Ali AM, Khodairy A, Marzouk AA, Nafady A, Nemr MTM (2024) Novel guanidine derivatives targeting leukemia as selective Src/Abl dual inhibitors: design, synthesis and anti-proliferative activity. Bioorg Chem 147:107410. 10.1016/j.bioorg.2024.107410 38688197 10.1016/j.bioorg.2024.107410

[43] Fadaly WAA, Mohamed FEA, Nemr MTM, Sayed AM, Khalil RG, Zidan TH (2024) Novel benzenesulfonamide derivatives as potential selective carbonic anhydrase IX, XII inhibitors with anti-proliferative activity: design, synthesis and in silico studies. Bioorg Chem. 10.1016/j.bioorg.2024.107881M 39396453 10.1016/j.bioorg.2024.107881

[44] Mohamed MFA, Shaykoon MShA, Abdelrahman MH, Elsadek BEM, Aboraia AS, G. El-Din A. A. Abuo-Rahma, (2017) Design, synthesis, docking studies and biological evaluation of novel chalcone derivatives as potential histone deacetylase inhibitors. Bioorg Chem. 10.1016/j.bioorg.2017.03.005 28346873 10.1016/j.bioorg.2017.03.005

[45] Shubhalaxmi L, Pathak K, Ananda KS, Bhat (2016) Synthesis of focused library of novel aryloxyacids and pyrazoline derivatives: molecular docking studies and antimicrobial investigation. Cogent Chem. 10.1080/23312009.2016.1141388

[46] Nikhil S, Rajvir S, Malik MS, Singh (2008) Synthesis and insecticidal activity of 2-[4-{4,5-di hydro-5-(substituted phenyl)-1*H*-pyrazol-3-yl}phenoxy] acetic acid hydrazides and related compounds. Ram Pesticide Res J 20(2):183–188

[47] Pommery N, Taverrne T, Telliez A, Goossens L, Charlier C, Pommery J, Goossens JF, Hossain R, Durant F, Heichart JP (2004) New COX-2/5-LOX inhibitors: apoptosis-inducing agents potentially useful in prostate cancer chemotherapy. J Med Chem 47:6195–6206. 10.1021/jm0407761 15566290 10.1021/jm0407761

[48] Soliman R (1979) Preparation and anti-diabetic activity of some sulfonylurea derivatives of 3,5-disubstituted pyrazoles. J Med Chem 22:321–325. 10.1021/jm00189a022 423216 10.1021/jm00189a022

[49] Roschek B Jr, Fink RC, Li D, McMichael M, Tower CM, Smith RD, Alberte RS (2009) Pro-inflammatory enzymes, cyclooxygenase 1, cyclooxygenase 2, and 5-lipooxygenase, inhibited by stabilized rice bran extracts. J Med Food 12(3):615–623. 10.1089/jmf.2008.0133 19627211 10.1089/jmf.2008.0133

[50] Yamamoto S (1992) Mammalian lipoxygenases: molecular structures and functions. Biochim Biophys Acta 1128:117–131 1420284 10.1016/0005-2760(92)90297-9

[51] Cho CH, Ogle CW (1979) Cholinergic-mediated gastric mast cell degranulation with subsequent histamine H1- and H2-receptor activation in stress ulceration in rats. Eur J Pharm 55(1):23–33. 10.1016/0014-2999(79)90144-4 10.1016/0014-2999(79)90144-4 436942

[52] RCSB Protein Data Bank. Available online: http://www.rcsb.org/pdb, (3LN1)

[53] RCSB Protein Data Bank. Available online: http://www.rcsb.org/pdb, (3V99)

[54] Mishra S, Rashm D (2019) In-vitro ADME studies of TUG-891, a GPR-120 inhibitor using Swiss ADME predictor. J Drug Deliv Ther 9(2):366–369. 10.22270/jddt.v9i3.2678

[55] Faidallah HM, Rostom SAF (2017) Synthesis, anti-inflammatory activity, and COX-1/2 inhibition profile of some novel non-acidic polysubstituted pyrazoles and pyrano[2,3-c]pyrazoles. Arch Pharm 350(5):1–17. 10.1002/ardp.201700025 10.1002/ardp.201700025 28370254

[56] Daina A, Michielin O, Zoete V (2017) SwissADME: a free web tool to evaluate pharmacokinetics, drug-likeness and medicinal chemistry friendliness of small molecules. Sci Rep 7:1–13. 10.1038/srep42717 28256516 10.1038/srep42717 PMC5335600

[57] Gandhi J, Khera L, Gaur N, Paul C, Kaul R (2017) Role of modulator of inflammation cyclooxygenase-2 in gamma herpes virus mediated tumorigenesis. Front Microbiol 8:538. 10.3389/fmicb.2017.00538 28400769 10.3389/fmicb.2017.00538 PMC5368278

[58] Okur ME, Karadağ AE, Özhan Y, Sipahi H, Ayla Ş, Daylan B, Kültür Ş, Demirci F. Demirci (2021) Anti-inflammatory, analgesic and in vivo-in vitro wound healing potential of the *Phlomis rigida* Labill. extract. J Ethnopharmacol 266:113408. 10.1016/j.jep.2020.113408 32979409 10.1016/j.jep.2020.113408

[59] Minhas R, Bansal Y, Bansal G (2020) Inducible nitric oxide synthase inhibitors: a comprehensive update. Med Res Rev 40:823. 10.1002/med.21636 31502681 10.1002/med.21636

[60] Sahlan M, Mahira KF, Pratami DK, Rizal R, Ansari MJ, Al-Anazi KM, Farah MA (2021) The cytotoxic and anti-inflammatory potential of *Tetragonula sapiens* propolis from Sulawesi on raw 264.7 cell lines. J King Saud Univ 33:101314. 10.1016/j.jksus.2020.101314

